# Extreme weather event attribution predicts climate policy support across the world

**DOI:** 10.1038/s41558-025-02372-4

**Published:** 2025-07-01

**Authors:** Viktoria Cologna, Simona Meiler, Chahan M. Kropf, Samuel Lüthi, Niels G. Mede, David N. Bresch, Oscar Lecuona, Sebastian Berger, John Besley, Cameron Brick, Marina Joubert, Edward W. Maibach, Sabina Mihelj, Naomi Oreskes, Mike S. Schäfer, Sander van der Linden, TISP Consortium, Viktoria Cologna, Niels G. Mede, Oscar Lecuona, Sebastian Berger, John Besley, Cameron Brick, Marina Joubert, Edward W. Maibach, Sabina Mihelj, Naomi Oreskes, Mike S. Schäfer, Sander van der Linden, Nor Izzatina Abdul Aziz, Suleiman Abdulsalam, Nurulaini Abu Shamsi, Balazs Aczel, Indro Adinugroho, Eleonora Alabrese, Alaa Aldoh, Mark Alfano, Innocent Mbulli Ali, Mohammed Alsobay, R. Michael Alvarez, Tabitha Amollo, Patrick Ansah, Denisa Apriliawati, Flavio Azevedo, Ani Bajrami, Ronita Bardhan, Keagile Bati, Eri Bertsou, Rahul Bhui, Olga Białobrzeska, Michal Bilewicz, Ayoub Bouguettaya, Katherine Breeden, Amélie Bret, Ondrej Buchel, Pablo Cabrera Alvarez, Federica Cagnoli, André Calero Valdez, Timothy Callaghan, Rizza Kaye Cases, Sami Çoksan, Gabriela Czarnek, Ramit Debnath, Sylvain Delouvée, Lucia Di Stefano, Celia Diaz-Catalàn, Kimberly C. Doell, Simone Dohle, Karen M. Douglas, Charlotte Dries, Dmitrii Dubrov, Malgorzata Dzimińska, Ullrich K. H. Ecker, Christian T. Elbaek, Mahmoud Elsherif, Benjamin Enke, Matthew Facciani, Antoinette Fage-Butler, Zaki Faisal, Xiaoli Fan, Christina Farhart, Christoph Feldhaus, Marinus Ferreira, Stefan Feuerriegel, Helen Fischer, Jana Freundt, Malte Friese, Albina Gallyamova, Patricia Garrido-Vásquez, Mauricio E. Garrido Vásquez, Olivier Genschow, Omid Ghasemi, Theofilos Gkinopoulos, Jamie L. Gloor, Ellen Goddard, Claudia González Brambila, Hazel Gordon, Dmitry Grigoryev, Lars Guenther, Håvard Haarstad, Dana Harari, Przemysław Hensel, Alma Cristal Hernández-Mondragón, Atar Herziger, Guanxiong Huang, Markus Huff, Mairéad Hurley, Nygmet Ibadildin, Mohammad Tarikul Islam, Tao Jin, Charlotte A. Jones, Sebastian Jungkunz, Dominika Jurgiel, Sarah Kavassalis, John R. Kerr, Mariana Kitsa, Tereza Klabíková Rábová, Olivier Klein, Hoyoun Koh, Aki Koivula, Lilian Kojan, Elizaveta Komyaginskaya, Laura M. König, Lina Koppel, Kochav Koren, Alexandra Kosachenko, John Kotcher, Laura S. Kranz, Pradeep Krishnan, Silje Kristiansen, André Krouwel, Toon Kuppens, Claus Lamm, Anthony Lantian, Aleksandra Lazić, Jean-Baptiste Légal, Zoe Leviston, Neil Levy, Amanda M. Lindkvist, Grégoire Lits, Andreas Löschel, Alberto López Ortega, Carlos Lopez-Villavicencio, Nigel Mantou Lou, Chloe H. Lucas, Kristin Lunz-Trujillo, Mathew D. Marques, Sabrina J. Mayer, Ryan McKay, Taciano L. Milfont, Joanne M. Miller, Panagiotis Mitkidis, Fredy Monge-Rodríguez, Matt Motta, Zarja Muršič, Jennifer Namutebi, Eryn J. Newman, Jonas P. Nitschke, Ntui-Njock Vincent Ntui, Daniel Nwogwugwu, Thomas Ostermann, Tobias Otterbring, Myrto Pantazi, Philip Pärnamets, Paolo Parra Saiani, Mariola Paruzel-Czachura, Michal Parzuchowski, Yuri G. Pavlov, Adam R. Pearson, Charlotte R. Pennington, Katerina Petkanopoulou, Marija B. Petrović, Dinara Pisareva, Adam Ploszaj, Ekaterina Pronizius, Karolína Pštross, Katarzyna Pypno-Blajda, Diwa Malaya A. Quiñones, Pekka Räsänen, Adrian Rauchfleisch, Felix G. Rebitschek, Gabriel Rêgo, James P. Reynolds, Joseph Roche, Jan Philipp Röer, Robert M. Ross, Isabelle Ruin, Osvaldo Santos, Ricardo R. Santos, Stefan Schulreich, Emily Shuckburgh, Johan Six, Nevin Solak, Leonhard Späth, Bram Spruyt, Samantha K. Stanley, Noel Strahm, Stylianos Syropoulos, Barnabas Szaszi, Ewa Szumowska, Mikihito Tanaka, Claudia Teran-Escobar, Boryana Todorova, Abdoul Kafid Toko, Renata Tokrri, Daniel Toribio-Florez, Manos Tsakiris, Michael Tyrala, Özden Melis Uluğ, Ijeoma Chinwe Uzoma, Jochem van Noord, Iris Vilares, Madalina Vlasceanu, Andreas von Bubnoff, Izabela Warwas, Tim Weninger, Mareike Westfal, Adrian Dominik Wojcik, Ziqian Xia, Jinliang Xie, Ewa Zegler-Poleska, Amber Zenklusen

**Affiliations:** 1 Department of Environmental Systems Science, ETH Zurich, Zurich, Switzerland; 2 Collegium Helveticum, Zurich, Switzerland; 3 Department of the History of Science, Harvard University, Cambridge, MA USA; 4 Federal Office of Meteorology and Climatology MeteoSwiss, Zurich-Airport, Switzerland; 5 Institute for Atmospheric and Climate Science, ETH Zurich, Zurich, Switzerland; 6 Department of Communication and Media Research, University of Zurich, Zurich, Switzerland; 7 Department of Psychobiology and Methodology, Faculty of Psychology, Universidad Complutense de Madrid, Madrid, Spain; 8 Institute of Sociology, University of Bern, Bern, Switzerland; 9 Department of Advertising and Public Relations, Michigan State University, East Lansing, MI USA; 10 Department of Psychology, University of Amsterdam, Amsterdam, the Netherlands; 11 Department of Psychology, University of Inland Norway, Lillehammer, Elverum Norway; 12 Centre for Research on Evaluation, Science and Technology, Stellenbosch University, Stellenbosch, South Africa; 13 Centre for Climate Change Communication, George Mason University, Fairfax, VA USA; 14 Centre for Research in Communication and Culture, Department of Communication and Media, Loughborough University, Loughborough, UK; 15 Department of Psychology, University of Cambridge, Cambridge, UK; 16 Institute of Malaysian and International Studies, National University of Malaysia, Bangi, Malaysia; 17 School of Collective Intelligence, Mohammed VI Polytechnic University, Ben Guerir, Morocco; 18 Department of Science and Technology Studies, Faculty of Science, Universiti Malaya, Kuala Lumpur, Malaysia; 19 ELTE Institute of Psychology, Eotvos Lorand University, Budapest, Hungary; 20 School of Psychology, University of Sheffield, Sheffield, UK; 21 Department of Economics, University of Bath, Bath, UK; 22 School of Psychology, University of Sussex/University of Amsterdam, Amsterdam, the Netherlands; 23 Department of Philosophy, Macquarie University, Sydney, Australia; 24 Department of Biochemistry, Faculty of Science, University of Dschang, Dschang, Cameroon; 25 Sloan School of Management, Massachusetts Institute of Technology, Cambridge, MA USA; 26 Linde Center for Science, Society, and Policy, Division of Humanities and Social Science, California Institute of Technology, Pasadena, CA USA; 27 Department of Physics, Egerton University, Egerton, Kenya; 28 Department of Communication, George Mason University, Fairfax, VA USA; 29 Department of Psychology, Universitas Islam Negeri Sunan Kalijaga, Yogyakarta, Indonesia; 30 Department of Interdisciplinary Social Science, University of Utrecht, Utrecht, the Netherlands; 31 National Institute of Science and Technology on Social and Affective Neuroscience, São Paulo, Brazil; 32 Museum of Natural Sciences ‘Sabiha Kasimati’, University of Tirana, Tirana, Albania; 33 Department of Architecture, University of Cambridge, Cambridge, UK; 34 Department of Biomedical Sciences, University of Botswana, Gaborone, Botswana; 35 Institute of Political Science, University of St Gallen, St Gallen, Switzerland; 36 Institute of Psychology, SWPS University, Warsaw, Poland; 37 Faculty of Psychology, University of Warsaw, Warsaw, Poland; 38 Cedars-Sinai Medical Center, Los Angeles, CA USA; 39 Computer Science Department, Harvey Mudd College, Claremont, CA USA; 40 Laboratoire de psychologie des Pays de la Loire, LPPL UR 4638, Nantes Université, Univ. Angers, Nantes, France; 41 Institute for Sociology of the Slovak Academy of Sciences, Bratislava, Slovakia; 42 Department of Scientific and Innovation Culture, Spanish Foundation for Science and Technology, Madrid, Spain; 43 Department of International and Political Sciences, University of Genoa, Genoa, Italy; 44 Institute of Multimedia and Interactive Systems, University of Lübeck, Lübeck, Germany; 45 Department of Health Law, Policy, and Management, Boston University School of Public Health, Boston, MA USA; 46 Institute for Sociology, Slovak Academy of Sciences, Staré Mesto, Slovakia; 47 Department of Psychology, Erzurum Technical University, Erzurum, Turkey; 48 Network for Economic and Social Trends, Western University, London, Ontario Canada; 49 Behavior in Crisis Lab, Institute of Psychology, Jagiellonian University, Cracow, Poland; 50 Cambridge Zero, University of Cambridge, Cambridge, UK; 51 LP3C (Psychology Laboratory), Université Rennes 2, Rennes, France; 52 TRANSOC, Complutense University of Madrid, Madrid, Spain; 53 Department of Cognition, Emotion, and Methods in Psychology, Faculty of Psychology, University of Vienna, Vienna, Austria; 54 Institute of General Practice and Family Medicine, University of Bonn, University Hospital Bonn, Bonn, Germany; 55 School of Psychology, University of Kent, Canterbury, UK; 56 Harding Center for Risk Literacy, University of Potsdam, Potsdam, Germany; 57 Center for Sociocultural Research, HSE University, Moscow, Russia; 58 Department of Labor and Social Policy, University of Lodz, Lodz, Poland; 59 School of Psychological Science and Public Policy Institute, University of Western Australia, Perth, Western Australia Australia; 60 Department of Management, Aarhus University, Aarhus, Denmark; 61 School of Psychology, University of Birmingham, Birmingham, UK; 62 Department of Economics, Harvard University, Cambridge, MA USA; 63 Department of Computer Science and Engineering, University of Notre Dame, Notre Dame, IN USA; 64 School of Communication and Culture, Aarhus University, Aarhus, Denmark; 65 a2i Programme of ICT Division and UNDP Bangladesh, Dhaka, Bangladesh; 66 Department of Resource Economics and Environmental Sociology, University of Alberta, Edmonton, Alberta Canada; 67 Department of Political Science and International Relations, Carleton College, Northfield, MN USA; 68 Faculty of Management and Economics, Ruhr-University Bochum, Bochum, Germany; 69 Department of Philosophy, Macquarie University, Sydney, New South Wales Australia; 70 LMU Munich School of Management, LMU Munich, Munich, Germany; 71 Leibniz Institut für Wissensmedien, Tübingen, Germany; 72 School of Social Work, Lucerne University of Applied Sciences and Arts, Lucerne, Switzerland; 73 Department of Psychology, Saarland University, Saarbrücken, Germany; 74 Department of Psychology, Universidad de Concepción, Concepción, Chile; 75 Institute for Management and Organization, Leuphana University, Lueneburg, Germany; 76 School of Psychology, University of New South Wales, Sydney, New South Wales Australia; 77 Institute for Climate Risk and Response, University of New South Wales, Sydney, New South Wales Australia; 78 Research Institute for Responsible Innovation, School of Management, University of St Gallen, St Gallen, Switzerland; 79 Department of Business Administration, Instituto Técnológico Autónomo de México, Mexico City, Mexico; 80 Department of Media and Communication, LMU Munich, Munich, Germany; 81 Department of Geography, University of Bergen, Bergen, Norway; 82 Centre for Climate and Energy Transformation, University of Bergen, Bergen, Norway; 83 Faculty of Data and Decision Sciences, Technion—Israel Institute of Technology, Haifa, Israel; 84 Faculty of Management, University of Warsaw, Warsaw, Poland; 85 Centro de Investigación y de Estudios Avanzados del Instituto Politícnico Nacional, Mexico City, Mexico; 86 Department of Media and Communication, City University of Hong Kong, Kowloon, Hong Kong; 87 Leibniz-Institut für Wissensmedien, Tübingen, Germany; 88 Department of Psychology, Eberhard Karls Universität Tübingen, Tübingen, Germany; 89 School of Education, Trinity College Dublin, Dublin, Ireland; 90 Department of Political Science and International Relations, KIMEP University, Almaty, Kazakhstan; 91 Department of Government and Politics, Jahangirnagar University, Dhaka, Bangladesh; 92 Department of Psychology, University of Minnesota, Minneapolis, MN USA; 93 School of Geography, Planning, and Spatial Sciences, University of Tasmania, Tasmania, Australia; 94 Institute of Political Science, University of Bamberg, Bamberg, Germany; 95 Institute of Political Science and Sociology, University of Bonn, Bonn, Germany; 96 Doctoral School of Social Sciences, Nicolaus Copernicus University, Toruń, Poland; 97 Hixon Center for Climate and the Environment, Harvey Mudd College, Claremont, CA USA; 98 Department of Public Health, University of Otago, Wellington, New Zealand; 99 Department of Journalism and Mass Communication, Lviv Polytechnic National University, Lviv, Ukraine; 100 Institute of Communication Studies and Journalism, Charles University, Prague, Czech Republic; 101 Center for Social and Cultural Psychology, Université Libre de Bruxelles, Brussels, Belgium; 102 Department of Political Science and International Relations, School of Sciences and Humanities, Nazarbayev University, Astana, Kazakhstan; 103 Department of Social Research, University of Turku, Turku, Finland; 104 INVEST Research Flagship Center, University of Turku, Turku, Finland; 105 Faculty of Life Sciences: Food, Nutrition and Health, University of Bayreuth, Kulmbach, Germany; 106 Department of Clinical and Health Psychology, Faculty of Psychology, University of Vienna, Vienna, Austria; 107 Division of Economics, Department of Management and Engineering, Linköping University, Linköping, Sweden; 108 Faculty of Polish and Classical Philology, University of Adam Mickiewicz, Poznań, Poland; 109 Department of Psychology, Ural Federal University, Yekaterinburg, Russia; 110 School of Psychology, Victoria University of Wellington, Wellington, New Zealand; 111 Department of Information Science and Media Studies, University of Bergen, Bergen, Norway; 112 Department of Communication Science and Political Science, Vrije Universiteit Amsterdam, Amsterdam, the Netherlands; 113 Faculty of Behavioural and Social Sciences, University of Groningen, Groningen, the Netherlands; 114 Laboratoire Parisien de Psychologie Sociale, Université Paris Nanterre, Nanterre, France; 115 Laboratory for Research of Individual Differences, University of Belgrade, Belgrade, Serbia; 116 School of Medicine and Psychology, Australian National University, Canberra, Australian Capital Territory Australia; 117 Uehiro Centre for Practical Ethics, University of Oxford, Oxford, UK; 118 Institut Langage et Communication, University of Louvain, Louvain, Belgium; 119 Departamento de Psicología, Universidad Peruana Cayetano Heredia, La Molina, Peru; 120 Department of Psychology, University of Victoria, Victoria, British Columbia Canada; 121 Harvard Kennedy School’s Shorenstein Center, Harvard University, Cambridge, MA USA; 122 Network Science Institute, Northeastern University, Boston, MA USA; 123 School of Psychology and Public Health, La Trobe University, Melbourne, Victoria Australia; 124 Department of Psychology, Royal Holloway, University of London, Egham, UK; 125 School of Psychological and Social Sciences, University of Waikato, Tauranga, New Zealand; 126 Department of Political Science and International Relations, University of Delaware, Newark, DE USA; 127 Office for Quality Assurance, Analyses and Reporting, Project EUTOPIA, University of Ljubljana, Ljubljana, Slovenia; 128 Department of Management and Supply Chain Studies, Nkumba University, Entebbe, Uganda; 129 Department of Biochemistry and Molecular Biology, University of Buea, Buea, Cameroon; 130 Communication Arts Programme, Bowen University, Iwo, Nigeria; 131 Department of Psychology and Psychotherapy, Witten/Herdecke University, Witten, Germany; 132 Department of Management, University of Adger, Kristiansand, Norway; 133 Department of Clinical Neuroscience, Karolinska Institutet, Stockholm, Sweden; 134 Institute of Psychology, University of Silesia in Katowice, Katowice, Poland; 135 Penn Center for Neuroaesthetics, University of Pennsylvania, Philadelphia, PA USA; 136 Institute of Medical Psychology, University of Tübingen, Tübingen, Germany; 137 Department of Psychological Science, Pomona College, Claremont, CA USA; 138 School of Psychology, Aston University, Birmingham, UK; 139 Department of Psychology, University of Crete, Rethymno, Greece; 140 Science Studies Laboratory, University of Warsaw, Warsaw, Poland; 141 Department of Psychology, University of the Philippines Diliman, Quezon City, Philippines; 142 Graduate Institute of Journalism, National Taiwan University, Taipei, Taiwan; 143 Max Planck Institute for Human Development, Berlin, Germany; 144 Social and Cognitive Neuroscience Laboratory, Mackenzie Presbyterian University, São Paulo, Brazil; 145 Institut des Géosciences de l’Environnement, University Grenoble Alpes, CNRS, IRD, Grenoble-INP, Grenoble, France; 146 Institute of Environmental Health, Faculty of Medicine, University of Lisbon, Lisbon, Portugal; 147 NOVA Institute of Communication, NOVA University of Lisbon, Lisbon, Portugal; 148 Department of Nutritional Sciences, University of Vienna, Vienna, Austria; 149 Department of Cognitive Psychology, Universität Hamburg, Hamburg, Germany; 150 Psychology Department, TED University, Ankara, Turkey; 151 Sociology Department, Vrije Universiteit Brussel, Brussels, Belgium; 152 School of Sustainability, Arizona State University, Tempe, AZ USA; 153 Faculty of Political Science and Economics, Waseda University, Tokyo, Japan; 154 Department of Civil Law, Faculty of Law, University of Tirana, Milto Tutulani, Tirana, Albania; 155 Centre for the Politics of Feelings, University of London, London, UK; 156 Division of Public Policy, Hong Kong University of Science and Technology, Hong Kong, Hong Kong; 157 School of Psychology, University of Sussex, Falmer, UK; 158 Molecular Haematology and Immunogenetics Laboratory, Department of Medical Laboratory Science, Faculty of Health Sciences and Technology, College of Medicine, University of Nigeria Nsukka, Nsukka, Nigeria; 159 Department of Environmental Social Sciences, Stanford Doerr School of Sustainability, Stanford, CA USA; 160 Faculty of Technology and Bionics, Rhine-Waal University, Kleve, Germany; 161 Faculty of Philosophy and Social Science, Nicolaus Copernicus University, Toruń, Poland; 162 School of Economics and Management, Tongji University, Shanghai, China; 163 School of Environment, Tsinghua University, Beijing, China

**Keywords:** Climate-change impacts, Climate-change policy, Attribution, Psychology

## Abstract

Extreme weather events are becoming more frequent and intense due to climate change. Yet, little is known about the relationship between exposure to extreme events, subjective attribution of these events to climate change, and climate policy support, especially in the Global South. Combining large-scale natural and social science data from 68 countries (*N* = 71,922), we develop a measure of exposed population to extreme weather events and investigate whether exposure to extreme weather and subjective attribution of extreme weather to climate change predict climate policy support. We find that most people support climate policies and link extreme weather events to climate change. Subjective attribution of extreme weather was positively associated with policy support for five widely discussed climate policies. However, exposure to most types of extreme weather event did not predict policy support. Overall, these results suggest that subjective attribution could facilitate climate policy support.

## Abstract (web summary)

Literature produced inconsistent findings regarding the links between extreme weather events and climate policy support across regions, populations and events. This global study offers a holistic assessment of these relationships and highlights the role of subjective attribution.

## Main

Climate change is increasing the frequency and intensity of extreme weather events (defined as an event that is rare at a particular place and time of year^1^), which puts a substantial proportion of the global population at physical and economic risk^1^. The cost of extreme weather events attributable to climate change is estimated at US$143 billion per year^2^. The impacts of extreme weather events are disproportionately felt in countries in the Global South^3^. Even though the Global South is at greater risk, attribution studies and social science research on human responses to such events overwhelmingly focus on countries and populations in the Global North^4–6^.

Mitigative action is needed to slow climate change and mitigate the impacts of extreme weather events. So far, global efforts have been insufficient, which calls for more stringent climate policies. Public support for climate policies is important because such support can drive governmental policy outputs^7^ and policymakers often respond to public demand for climate policies^8^.

The psychological distance of climate change (that is, the perception that climate change is spatially, temporally and socially distant) may help explain societal inaction on this issue^9^. If so, public awareness and understanding of climate change may increase as more people experience extreme weather events for themselves^10–15^. However, previous studies on the relationship between experiencing extreme weather events and climate change action and beliefs have produced inconsistent findings. In particular, some studies have found that experiencing extreme weather events increases climate change belief^16^, concern^11,17–19^, support for climate policies and green parties^17,20–23^, and climate change adaptation^24^, while other studies found no relationship^6,25–27^. Studies using aggregate objective measures of exposure to and impacts of extreme weather events often find no effect of extreme weather experience on climate change attitudes^25,26,28^. For example, one US study found that living in an area with higher fatalities from extreme weather events was associated with perceiving more climate risks^29^, while another US study found that fatalities from extreme weather events were not associated with opinions about climate change^30^. However, these studies used different definitions and measurements of extreme weather events, and these extreme weather events were compared with different psychological and behavioural outcomes^27^. Further, most studies have focused on a single country^31^ or a single type of extreme weather event (for example, heatwaves), which limits the comparability of the impacts of different types of extreme weather event. This limitation is considerable, as a meta-analysis found notable differences in effect sizes depending on the type of extreme weather event^32^.

The inconsistency of previous studies might also be explained by another important factor: whether people attribute the extreme weather event to climate change^6,11,31,33–35^. Recent studies support this hypothesis: people who attribute extreme weather events to climate change are more likely to perceive climate change as a risk and to report engaging in mitigation behaviour^36,37^. For example, a study in the United Kingdom found that the subjective attribution of floods to climate change is a necessary condition for the experience of floods to translate into climate change threat perception^36^. However, no cross-country evidence exists on the subjective attribution of extreme weather events to climate change.

## Current study

We combined natural and social science approaches to examine how extreme weather events and their attribution to climate change relate to support for widely discussed climate change mitigation policies across 68 countries (*N* = 71,922). This study employed an interdisciplinary design by triangulating data on exposed populations computed using the probabilistic CLIMADA risk modelling platform^38,39^ with global survey data on subjective attribution of extreme weather events and support for climate policies collected in the Trust in Science and Science-related Populism (TISP) study^40^. We used a standardized metric to comparatively assess the relationship between the size of exposed populations to several extreme weather events—river floods, heatwaves, European winter storms, tropical cyclones, wildfires, heavy precipitation and droughts—and climate policy support. Specifically, we modelled how many people in a country were exposed to extreme weather events over the past few decades relative to the total population. We referred to this as the ‘exposed population’ (see Online Methods).

Our preregistered study addressed the following research questions: (1) Does exposure to extreme weather events on the population level relate to climate policy support? (2) Do subjective attribution and exposed population have an interactive effect on policy support? In addition, we addressed the following non-preregistered questions: (1) What is the level of public support for five climate policies across countries? (2) To what degree do people attribute extreme weather events to climate change across countries (subjective attribution) and is subjective attribution related to policy support?

We hypothesized that people who live in countries with higher exposure would show stronger support for mitigative climate policies, and that the relationship between exposed population and policy support would be stronger for individuals with higher subjective attribution. We also hypothesized that the relationship between exposed population and policy support is associated with people’s income and residence area (urban vs rural), which might relate to their adaptation potential to extreme events. Note that not all preregistered questions are addressed in this paper.

## Support for climate policies

**Fig. 1:**
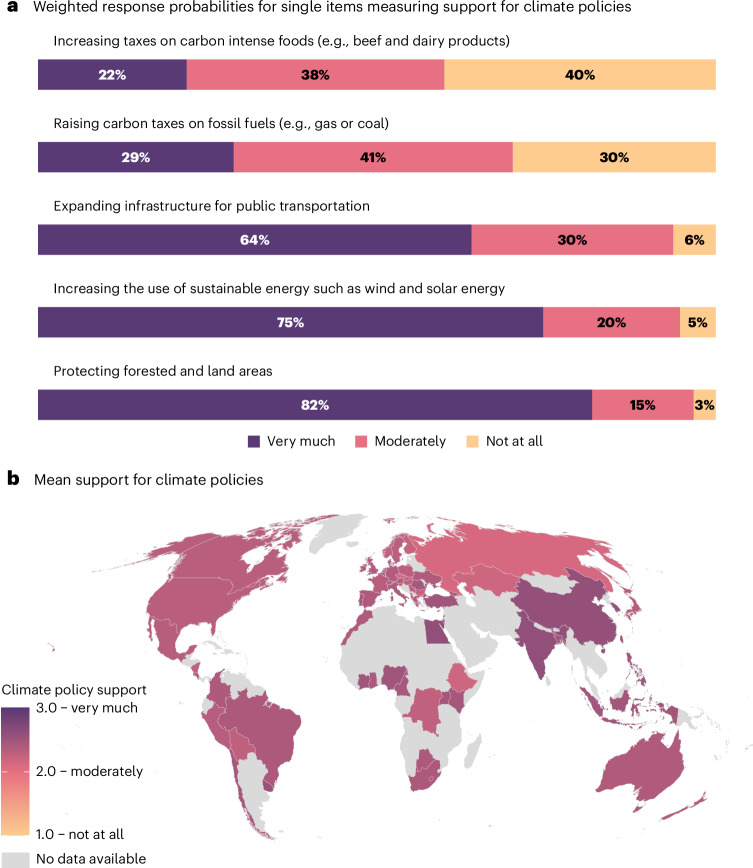
Global evidence of the support for climate policies. **a**, Weighted response probabilities for single items measuring support for climate policies. **b**, Mean support for climate policies in 66 countries (climate policy support was not measured in Argentina and Malaysia). Participants were asked: “Please indicate your level of support for the following policies.” Response option ‘not applicable’ is not shown. No data were available for countries shaded in light grey.

We assessed support for the following five climate policies with a 3-point scale (1 = not at all, 2 = moderately, 3 = very much): Increasing taxes on carbon-intense foods, raising taxes on fossil fuels, expanding infrastructure for public transportation, increasing the use of sustainable energy, and protecting forested and land areas. In line with previous research, increasing carbon taxes received the lowest support^41,42^, with only 22% and 29% of people, respectively, indicating they very much support increased taxes on carbon-intensive foods and fossil fuels (Fig. 1a). Protecting forested and land areas, by contrast, was a popular policy option, with 82% supporting it very much and only 3% not supporting it at all. The second most-supported policy was increasing the use of sustainable energy, with 75% supporting it very much, and only 5% not supporting it at all. For further analyses, we combined responses to the five policy options into an index (α = 0.61; see factor analysis in Supplementary Table 12 and non-preregistered analyses with policy subscales in Supplementary Fig. 7).

A clear majority supported climate policies in all countries (global mean (*M*) = 2.37, s.d. = 0.43 on a scale from 1 = Not at all, 2 = Moderately and 3 = Very much). These findings are in line with a previous study showing that 89% of participants demand intensified political action on climate change^43^. We calculated mean support by averaging participants’ support for five policies (see Online Methods and Fig. 1). This mean value is representative in terms of gender, age and education due to post-stratification weighting (see Online Methods). We found strong differences in support across countries and policies (Fig. 1b). Support for climate policies was particularly high in African and Asian countries, average in Australia, Costa Rica and the United Kingdom, and below the global average in several European countries, such as Czechia, Finland and Norway (Supplementary Figs. 1–6). Non-preregistered analyses comparing our aggregate measure with policy support subscales (that is, support for taxes, support for green transition) can be found in Supplementary Fig. 7. Our results for the aggregate measure and policy subscales were mostly consistent.

Participants who identified as men, were younger, more religious, had higher education, higher income, left-leaning politics and who lived in urban areas were more likely to support climate policies (Supplementary Tables 1–7 and Fig. 8), in line with previous studies^44,45^.

## Subjective attribution

Participants indicated subjective attribution by rating the degree to which they believed that climate change has increased the impact of six extreme weather events—droughts, heatwaves, wildfires, heavy rain, floods, heavy storms—in their country over the past decades (1 = Not at all, 5 = Very much). Responses to the six items were mean averaged (α = 0.92). Globally, subjective attribution of extreme weather events to climate change was well above the scale midpoint in all countries (*M* = 3.80, s.d. = 1.02). In line with a previous study^36^, non-preregistered analyses showed that subjective attribution was positively related to identifying as a woman, being older, more religious, having higher education and higher income, living in an urban (vs rural) area and self-identifying as politically liberal and left-leaning (Supplementary Table 8).

**Fig. 2:**
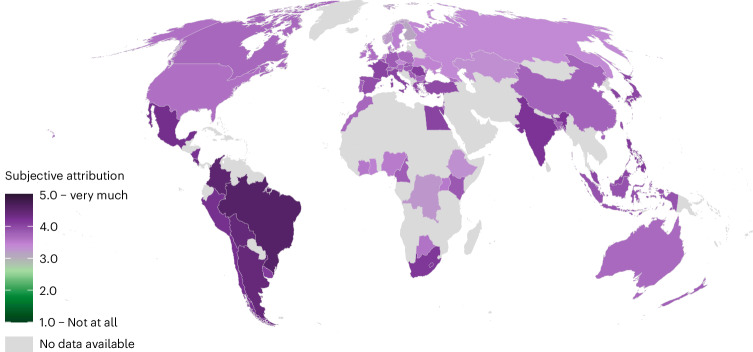
Subjective attribution of extreme weather events to climate change (mean index) over the past decades. Data from 67 countries. Subjective attribution was not assessed in Albania. No data were available for countries shaded in light grey.

There was little variation in subjective attribution across extreme event types. Subjective attribution appeared relatively lower for wildfires (*M* = 3.67, s.d. = 1.28) and higher for heatwaves (*M* = 3.94, s.d. = 1.16). However, subjective attribution varied across global regions (Fig. 2). Participants in South American countries most strongly agreed that the occurrence of extreme weather events has been affected by climate change over the past decades, especially in Brazil and Colombia (Supplementary Fig. 9). Subjective attribution was lowest in Northern European and African countries (Supplementary Fig. 9). Lower subjective attribution in African countries could be explained by the fact that climate change awareness and belief in human-caused climate change are still relatively low across African countries^46^.

## Exposed population and policy support

**Fig. 3:**
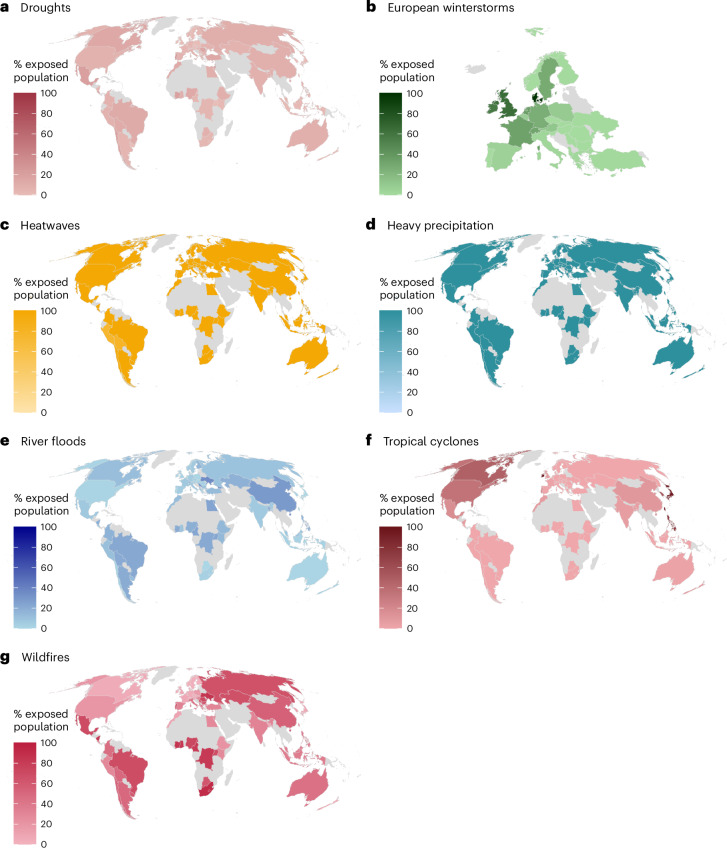
Exposed population across countries over the past few decades. Exposed population refers to the average annual proportion of a country’s total population exposed to a specific weather-related hazard and averaged over the past few decades. The exact time frame varies slightly across events. Exposed population is modelled for the 68 countries included in the survey. **a**, Exposed population to droughts. **b**, Exposed population to European winter storms. **c**, Exposed population to heatwaves. **d**, Exposed population to heavy precipitation. **e**, Exposed population to river floods. **f**, Exposed population to tropical cyclones. **g**, Exposed population to wildfires. No data were available for countries shaded in light grey.

The size of the exposed population varied by the type of extreme event (Fig. 3). While almost all the sampled populations were exposed to heatwaves and heavy precipitation over the past decades at least once, fewer populations had been exposed to droughts, wildfires and floods. Our fully anonymous data did not allow geospatially matching participants to certain areas where extreme events occurred; we therefore do not know whether participants were personally exposed to those events and cannot test whether exposure at the individual level relates to policy support. However, we can reliably estimate whether exposure at the population level relates to policy support.

We investigated whether exposure at the country level and subjective attribution of extreme events at the individual level were associated with stronger climate policy support. Since we were interested in studying how the relationships vary between different types of extreme weather event and policy support, we ran seven blockwise multilevel regression models—one for each type of extreme weather event—predicting an index of climate policy support. Because participants were clustered within countries, our models included random intercepts across countries. Step 1 of the blockwise regression included socio-demographic variables and exposed population. In Step 2, we added subjective attribution for the specific event and three interaction terms: exposed population × subjective attribution, exposed population × income and exposed population × residence area.

**Fig. 4:**
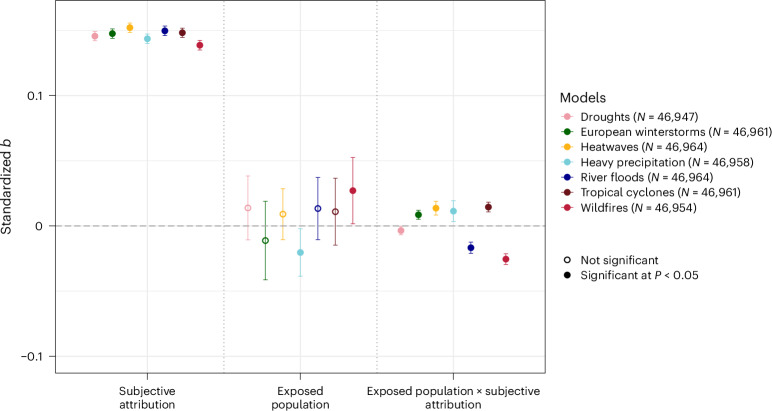
Weighted blockwise multilevel models predicting climate policy support. Summary of seven multilevel models, one for each type of extreme weather event, with random intercepts across countries predicting climate policy support and controlling for socio-demographic variables and two additional interaction terms. Models include data from 65 countries. Error bars denote 95% confidence intervals. Circles denote standardized estimates. Filled circles denote significant effects at *P* < 0.05. Exact *P* values for non-significant effects of exposed population: droughts: *P* = 0.275; European winter storms: *P* = 0.466; heatwaves: *P* = 0.369; river floods: *P* = 0.278; tropical cyclones: *P* = 0.409. Full models for each event type can be found in Supplementary Tables 1–7.

Belief that climate change has impacted local extreme weather events predicted support for climate policy (Fig. 4). Random effects models show that the relationship between subjective attribution and policy support was significantly stronger in North America, Australia and in several European countries than the mean global effect, and significantly weaker in Peru and South Africa (Supplementary Figs. 10–16).

For five out of the seven extreme weather events, exposed population size did not predict policy support (Fig. 4 and Supplementary Tables 1–7). However, people in countries more exposed to wildfires were more supportive of climate policies (Supplementary Table 5). Conversely, people in countries more exposed to heavy precipitation were less supportive of climate policies (Supplementary Table 3). We conducted additional exploratory, non-preregistered robustness checks to investigate whether exposed population and land area, as well as exposed population and climate change belief at the country level had an interactive effect on policy support. Since climate change belief was not assessed in this study, we relied on country-level data from another study^47^, available for 48 countries included in this study. The relationship between exposure to heavy precipitation/wildfires and policy support was no longer statistically significant when controlling for beliefs and land area, while the relationship between subjective attribution and policy support remained significant (Supplementary Fig. 17). Therefore, the relationship between exposure to wildfires/heavy precipitation and policy support should be interpreted with caution.

**Fig. 5:**
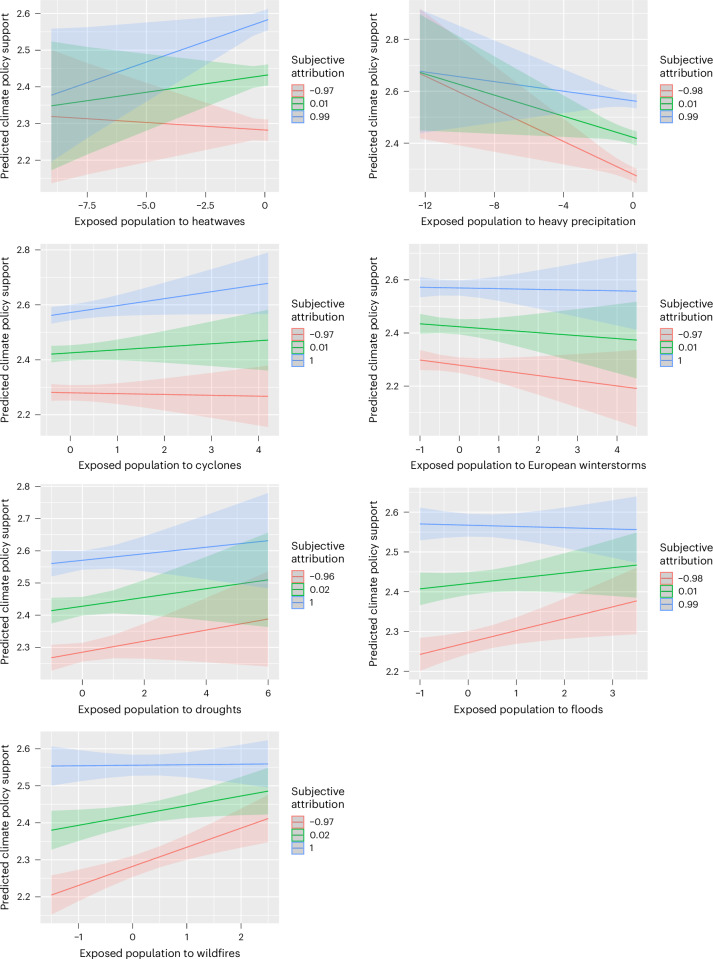
Interactions between subjective attribution and exposed population to extreme weather events on climate policy support. The lines represent varying levels of subjective attribution at −1s.d, the mean and +1 s.d., with shaded regions indicating 95% confidence intervals. The *x* axis shows the standardized exposed population size.

We tested whether exposed population size and subjective attribution interacted to predict policy support, as investigated in previous studies^33,36,37^. We found that the relationship between exposed population and policy support was stronger for participants with higher attribution of heatwaves and tropical cyclones, whereas the relationship between exposed population and policy support was weaker for participants with higher attribution of heavy precipitation and European winter storms. However, we found the opposite interaction effect for river floods, droughts and wildfires: as subjective attribution increases, the relationship between exposed population and policy support weakens. In other words, for individuals with high subjective attribution, support for policies is already high and less dependent on exposure to these extreme events. In contrast, for individuals with low subjective attribution, support for policies increases with higher exposure to droughts, floods and wildfires (Fig. 5).

These findings are in tension with the results of previous studies, which reported a positive moderation effect for flooding^36^, a negative moderation effect for hurricanes^33^ and no moderation effect for wildfires^37^.

## Interaction effects with income and residence area

Our seven multilevel models each included interaction effects for exposed population × income and exposed population × residence area. We found significant interactions with small effect sizes for river floods and wildfires, but not for any other events. For river floods, we found a negative interaction effect with income and a positive interaction with urban areas (Supplementary Table 4). This indicates that the relationship between exposed population size and policy support was stronger for individuals with lower income as well as for individuals who live in urban areas. For wildfires, we found a positive statistical effect for income, meaning that the relationship between exposed population and policy support was stronger for richer individuals (Supplementary Fig. 18).

## Discussion

This study provides global evidence that subjective attribution of extreme weather events to climate change is associated with greater policy support for climate mitigation. Overall, different extreme weather events appear to have different relationships with climate policy support. This pattern highlights the importance of comparative analyses that consider different types of event.

We additionally provide evidence that subjective attribution is high, and particularly so in Latin America. This might be explained by the fact that belief in human-caused climate change and self-reported personal experience of extreme weather events are high in Latin America^48^, and that people in Latin American countries were among the most likely to report that climate change will harm them and future generations a great deal and that climate change should be a high priority for their government^49^. The finding that the relationship between subjective attribution and policy support was weaker in some Latin American countries might therefore be due to a ceiling effect

In line with previous studies^36^, we also found that subjective attribution interacts with exposure to European winter storms, heatwaves, heavy precipitation and tropical cyclones to predict climate policy support. Mere exposure to extreme weather events might therefore not suffice to increase policy support unless individuals link these events to climate change^30^. While larger exposure to extreme events was not found to be related to policy support (except for wildfires), we cannot rule out that changes in the frequency of extreme weather events over time might be sufficient to shift support. Nevertheless, our data suggest that if individuals attribute extreme weather events to climate change, support for climate policies is higher regardless of whether the events are more frequent. The reverse causal relationship is also possible: people who are supportive of climate policies are more likely to attribute extreme weather to climate change. Longitudinal panel studies are needed to investigate the nature and direction of this relationship.

These findings might also help explain previous inconsistent results on the relationship between extreme weather event experience and mitigation behaviour. Few of these studies assessed whether participants linked these events to climate change, therefore missing a key controlling variable. Consequently, we strongly recommend that future studies assess subjective attribution. We found a negative relationship between exposed population to heavy precipitation and policy support in our preregistered model. Subjective attribution was relatively low for heavy precipitation. This corroborates previous findings that people often fail to link extreme rainfall with climate change^10^. In line with this argument, a media analysis that investigated themes in climate change coverage in 10 countries (2006–2018) found that media reporting on extreme weather events mostly focused on weather anomalies, as well as fires, hurricanes and storms^50^. Countries more exposed to heavy precipitation might therefore be less willing to support climate policies because they are less likely to link those events to climate change. Our moderation analyses show that the negative effect of heavy precipitation exposure on policy support is strongest for people with low subjective attribution. This further highlights the need for more research on climate change communication on types of extreme weather event that are not typically associated with climate change, such as heavy precipitation, as these events might serve as ‘teachable moments’^15^. However, it should be noted that the relationship between exposure to heavy precipitation and policy support was no longer significant in our exploratory analyses that included the interactions of exposed population with land area and climate change belief. This finding should therefore be interpreted with caution.

Wildfires are the only type of extreme weather event that positively predicts climate policy support when controlling for subjective attribution, although this effect was no longer significant in models that included interaction effects for exposure with land area and climate change belief. Several previous studies similarly reported a positive relationship between wildfire exposure and climate policy support^23,37,51,52^. This positive relationship could be explained by the fact that wildfires often result in extensive and visible damage^51^, and are linked to personal health concerns due to smoke exposure^53^. Another study found that among Australian adults who directly experienced wildfires, 45% increased individual climate activism, providing further evidence of the effects of wildfires on behavioural intentions^54^.

Contrary to our hypothesis, the relationship between exposed population and policy support was weaker for individuals with higher subjective attribution of droughts, floods and wildfires. One possible explanation is that these three types of extreme weather event allow for management strategies that can directly reduce the hazard itself, such as man-made flood protections, irrigation systems, prescribed burn-offs and land-use policies. Therefore, people may be more likely to support policies pertaining to law enforcement or economic regulations instead of climate change mitigation^55,56^. In contrast, although heavy precipitation, storms and heatwaves are exacerbated by climate change and can be mitigated by addressing it, once they occur, we can only manage their impacts, not prevent their occurrence. Future research should investigate these interactions and explore the possibility that the size of the exposed population moderates the relationship between subjective attribution and policy support, rather than subjective attribution moderating the effect between the size of the exposed population and policy support.

Our measure of exposed population has strengths and limitations. While the standardized metric of exposed population allows the comparison of the impacts of different events across countries, it is a relative measure (that is, to a country’s total population) and does not reflect the severity of exposure or the potential for individuals to be repeatedly exposed to different events. Further, the measure does not consider the exposure to compound events^57^, that is, when two or more events occur in an interacting combination. No conclusions can be drawn as to whether the participants in the study were directly exposed to these events. This measure therefore reflects the broader population-level exposure to these events, rather than individual-level exposure. The data cannot speak to whether exposure at the individual level relates to policy support. However, it can be reliably concluded that exposure at the population level did not relate to policy support. Some extreme weather events are less likely to be experienced directly (for example, floods or hurricanes), but they still receive widespread media coverage. The approach of analysing exposure at the population level therefore allows the study of effects that go beyond individual exposure to events. It should be noted that for some extreme weather events (for example, heatwaves and heavy precipitation), variance was very low, given that most people were affected by these events at some points over the past few decades (Supplementary Table 9).

Since the measure of exposed population included the past few decades, the estimates here are probably conservative for the effects of exposure. Researchers have found that temporal proximity of an event matters for climate change concern: the more recent an event, the larger the impact on climate change concern^18^. Since some of these events occur infrequently (for example, tropical cyclones), longer time frames such as in this study have the advantage that they allow the comparison of the effects of several different events in a global context^58^.

With the use of a measure of exposure to extreme weather events at the population level, this article finds that subjective attribution predicts climate policy support, while exposure to five out of the seven extreme events considered in this study does not predict policy support. Overall, ensuring subjective attribution might be an important way to increase support for climate policies^37^. Experimental research could focus on finding effective communication strategies to increase subjective attribution among the public to help develop causal models (for example, ref. ^59^). Extreme weather events are increasingly linked to climate change in news and social media^50,60–63^, but more research is needed to study communication of extreme weather events and their attribution in the Global South^62,64^.

## Methods

### Dataset

This study relies on the dataset collected for the TISP Many Labs study^40^. Detailed information on the data collection strategy can be found in ref. ^65^. Participants were asked to carefully read a consent form (approved under IRB protocol number IRB22-1046), which included some general information about the study and the anonymity of the data. Only participants who consented to participating in the study were allowed to proceed with the study.

### Sample and weighting

Data were collected in surveys that used quotas for age (five bins: 20% 18–29 years, 20% 30–39 years, 20% 40–49 years, 20% 50–59 years, 20% 60 years and older) and gender (two bins: 50% men, 50% women). To generate models with parameters that are representative for target populations in terms of gender, age and education, and have more precise standard errors, we used post-stratification weights. Specifically, we computed post-stratification weights at country level, sample size weights for each country, post-stratification weights for the complete sample, and rescaled post-stratification weights for multilevel analyses.

### Main measures included in the questionnaire

#### Climate policy support

Participants were asked: “Many countries have introduced policies to reduce carbon emissions and mitigate climate change. This can include the implementation of laws aiming to reduce greenhouse gases, for example. Please indicate your level of support for the following policies: 1) Raising carbon taxes on gas and fossil fuels or coal, 2) Expanding infrastructure for public transportation, 3) Increasing the use of sustainable energy such as wind and solar energy, 4) Protecting forested and land areas, 5) Increasing taxes on carbon intense foods (for example, beef and dairy products).” Response options ranged from 1 = Not at all, 2 = Moderately, 3 = Very much, and 4 = Not applicable. Response option 4 was coded as missing for the analyses.

#### Subjective attribution

Participants were asked: “The next questions are about climate change and weather events. When you answer them, please think about your country. To what extent do you think that climate change has increased the impact of the following weather events over the last decades? 1) Floods, 2) Heatwaves, 3) Heavy storms, 4) Wildfires, 5) Heavy rain, 6) Droughts.” Response options ranged from 1 = Not at all, to 5 = Very much.

See ref. ^65^ for a detailed overview of the other measures.

#### Analyses

We submitted a detailed preregistration including research questions, hypotheses and an analysis plan to OSF (10.17605/OSF.IO/G23A7) before data collection on 15 November 2022.

To estimate the relationships between subjective attribution, exposed population and three interaction terms (exposed population × subjective attribution; exposed population × income log (US$); exposed population × residence area (urban vs rural)), we used blockwise multilevel regression models with random intercepts across countries. In addition, we computed models with random effects to estimate how the effects of subjective attribution on climate policy support varied across countries. We scaled all independent variables by country means and country s.d.s, except for the country-level variable ‘exposed population’, which we scaled with grand means and grand s.d.s.

We estimated the reliability of our two scales: subjective attribution and climate policy support. Scale reliability of subjective attribution in the global sample was very high, with Cronbach’s alpha = 0.92 and omega = 0.92. An overview of the reliability of subjective attribution across 67 countries (ranging from omega = 0.74 to omega = 0.95) can be found in Supplementary Table 10. Scale reliability of climate policy support in the global sample was acceptable, with Cronbach’s alpha = 0.61 and omega = 0.62. An overview of the reliability of climate policy support across 66 countries (ranging from omega = 0.40 to omega = 0.75) can be found in Supplementary Table 11. To further assess the robustness of our policy support scale, we ran a polychoric parallel analysis with principal axis factoring to inspect how many factors should be retained for an exploratory factor analysis (EFA). The parallel analysis determined that two factors should be kept for an EFA. We therefore ran an EFA with unweighted least squares factoring and promax oblique rotation to inspect two factor loadings (Supplementary Table 12). Our items clearly loaded on two factors, with items relating to the expansion of public transport, protected areas and increasing renewable energy loading on Factor 1 (labelled as ‘Green transition’) and the two items related to increasing taxes on meat and dairy and fossil fuels loading on Factor 2 (labelled as ‘Taxes’). The Taxes subscale had good internal reliability (omega = 0.73). The Green transition subscale had moderate, but still acceptable reliability (omega = 0.61), comparable with the reliability of the aggregate scale (omega = 0.62).

We further conducted three non-preregistered robustness checks. Specifically, we examined whether our results are robust to the inclusion of an interaction between land area of countries (in square kilometres) and exposed population, an interaction between country-level climate change belief and exposed population, and across the two climate policy support subscales (Taxes and Green Transition). Data on climate change belief were retrieved from the Climate Many Labs study as processed by Our World in Data^66^, while data on land area were retrieved from multiple sources compiled by World Bank (2024) and processed by Our World in Data^67^. Data on land area for Taiwan was retrieved from ref. ^68^. The term ‘country’ in this Article refers to both sovereign states and territories not recognized as such.

### Impact model CLIMADA

In this study, we used the open-source, probabilistic CLIMADA (CLIMate ADAptation) risk modelling platform^38,39^ for the spatially explicit computation of exposed population from different hazards on a grid at 150 arc-seconds (~4.5 km at the equator) resolution. CLIMADA was designed to simulate the interaction of climate and weather-related hazards, the exposure of assets or populations to this hazard, and the specific vulnerability of exposed infrastructure and people in a globally consistent fashion. The platform has been developed and maintained as a community project, and the Python 3 source code is openly available under the terms of the GNU General Public License (v.3)^39^.

### Exposure

We used the Gridded Population of the World (GPW) dataset v.4.11, published in 2020 (CIESIN, 2018)^69^, to map population exposure across the 68 countries. The GPW dataset was chosen for its high spatial resolution and its comprehensive and consistent coverage, providing population count estimates at a granularity of 30 arc-seconds (~1 km at the equator), which we aggregated to match the 150-arc-second resolution used in our risk model.

### Hazards

Seven types of extreme weather event were analysed in this study: droughts, river floods, heatwaves, heavy precipitation, tropical cyclones, wildfires and European winter storms, which form the input hazard layer in our risk model. We computed the exposed population to these events. Detailed information on the definition of each event, data sources, the years covered and other relevant details for each type of extreme weather event are provided in Supplementary Table 13.

Each hazard in this study was defined on the basis of its unique characteristics and the potential impact it has on the exposed population, with the chosen underlying datasets ensuring consistent coverage across all countries involved. Some of these hazards were evaluated in an event-based perspective (for example, tropical cyclones, wildfires), while others were assessed as annually aggregated measures (for example, river floods, heatwaves). Hazards were inferred either from historical records (tropical cyclones, European winter storms, wildfires), climate reanalyses of a reference period (heatwaves, heavy precipitation) or historical climate modelling (droughts, river floods). In instances where multiple (climate) models contribute to the hazard modelling, we computed the multimodel median impact on the exposed population.

For drought, we utilized a ‘long-term’ definition based on soil moisture^70^, a methodology that primarily captures agricultural impacts, potentially leading to indirect effects on populations. Furthermore, the dataset provides annual maxima, without representing single drought events, which potentially limits the depth of our risk analysis for certain areas.

In the case of river floods, the datasets used in this study represent large rivers and fluvial floods, while coastal or pluvial floods are not included^70,71^. We note that ‘heavy precipitation’ as a different hazard may serve as a proxy for pluvial or flash floods. Besides, there was a potential overestimation of affected areas due to the methodology of considering full grid cells as affected.

For heatwaves and extreme precipitation events, we characterized the hazards on the basis of deviations from the 20-year reference period 1980–1999. We utilized ERA-5 reanalysis data to display observed trends as changes between the reference period and the more recent 20-year period 2000–2019^72^. Finally, changes were displayed as the multimodel median.

Wildfires of the historical period 2000–2019 were assessed using satellite imagery to derive thermal anomalies. A grid cell was considered affected if the temperature exceeded 300 K^73^. The historical period is determined by the data availability through the MODIS satellite mission. The approach does not distinguish between intentional and unintentional fires, and the dataset captures gridpoint-specific annual maxima only.

Finally, in our preregistration, we broadly categorized tropical cyclones and European winter storms under the umbrella term ‘storms’. Typically, tropical cyclones prevail in tropical and subtropical regions, while our modelled winter storms are predominantly observed in Europe. Given their distinct geographical occurrences, the impacts of these two storm types can be considered additive or complementary. However, tropical cyclone impacts in higher latitudes, where storms often undergo extratropical transition (for example, Sandy in 2012, Dorian in 2019, Fiona in 2020), were included in the tropical cyclone category due to their origin. While this classification ensured consistency with our framework, modelling these exposures carries higher uncertainty compared with the tropics and subtropics. In addition, storm impacts are expressed relative to population size, which may lead to disproportionately high exposure percentages in regions with low population density compared with densely populated areas experiencing similar storm frequencies. We relied on historical records to assess the impacts of both storm hazards^74,75^, and readers should interpret the results for higher latitudes with these considerations in mind.

### Definition of exposed population

In this study, we defined ‘exposed population’ as the average annual proportion of a country’s total population exposed to a specific weather-related hazard within a given time period. An overview of time periods can be found in Supplementary Table 13. This was calculated by summing the number of individuals in each 150-arc-second grid cell who have experienced the hazard at least once during the study period and then dividing this sum by the country’s total population, based on the GPW dataset. Therefore, this metric is relative and does not reflect the severity of exposure or the potential for individuals to be repeatedly impacted by different events. In addition, in large countries such as the United States, different hazards may affect different regional populations (for example, wildfires on the West Coast versus tropical cyclones in the East) which, unfortunately, is not captured in our country-level aggregation. The exposed population is presented as a percentage of the total population, providing a standardized measure for comparative analysis across the 68 countries included in our study.

### Reporting summary

Further information on research design is available in the Nature Portfolio Reporting Summary linked to this article.

## Online content

Any methods, additional references, Nature Portfolio reporting summaries, source data, extended data, supplementary information, acknowledgements, peer review information; details of author contributions and competing interests; and statements of data and code availability are available at 10.1038/s41558-025-02372-4.

## Supplementary information

Supplementary Information Supplementary Figs. 1–18 and Tables 1–13.

Reporting Summary

## Data Availability

The dataset on subjective attribution and policy support analysed during the current study is available in the Open Science Framework (OSF) repository at 10.17605/OSF.IO/5C3QD (ref. ^76^). The dataset on exposed populations to extreme weather events generated and analysed during the current study is available in OSF at 10.17605/OSF.IO/G23A7 (ref. ^77^).

## Code availability

The analysis code is available in OSF at 10.17605/OSF.IO/G23A7 (ref. ^77^).
