# Supplementary material for: Extreme weather event attribution predicts climate policy support across the world
Source: Nat Clim Chang. 2025 Jul 1;15(7):725–35. doi: 10.1038/s41558-025-02372-4 (PMC12237696; doi:10.1038/s41558-025-02372-4)
Supplement: Supplementary file 1 — Supplementary Figs. 1–18 and Tables 1–13. [file 41558_2025_2372_MOESM1_ESM.pdf]

nature climate change

Supplementary information https://doi.org/10.1038/s41558-025-02372-4

Extreme weather event attribution predicts climate policy support across the world

In the format provided by the authors and unedited

Table of Contents

Figure S1. Mean and standard errors of climate policy support (mean index), $N = 67{,}626$ 3

Figure S2. Mean and standard errors of support for taxes on carbon intense foods, $N = 64{,}038$ 4

Figure S3. Mean and standard errors of support for fuel taxes, $N = 64{,}203$ 5

Figure S4. Mean and standard errors of support for public transport, $N = 64{,}732$ 5

Figure S5. Mean and standard errors of support for sustainable energy, $N = 64{,}278$ 6

Figure S6. Mean and standard errors of support for protecting forested and land areas, $N = 63{,}566$........... 8

Figure S7. Weighted linear multilevel models comparing effects across policy measures (aggregate, taxes, green transition) for each extreme weather event (random intercepts across countries). 9

Figure S8. Effect of male gender on climate policy support across countries. 10

Figure S9. Mean and standard errors of subjective attribution across countries, $N = 69{,}163$.................... 11

Figure S10. Random effects for subjective attribution (heatwaves) on policy support 12

Figure S11. Random effects for subjective attribution (droughts) on policy support................................ 13

Figure S12. Random effects for subjective attribution (river floods) on policy support 14

Figure S13. Random effects for subjective attribution (wildfires) on policy support............................... 15

Figure S14. Random effects for subjective attribution (tropical cyclones) on policy support................... 16

Figure S15. Random effects for subjective attribution (heavy precipitation) on policy support 17

Figure S16. Random effects for subjective attribution (European winterstorms) on policy support.......... 18

Figure S17. Comparison of three models as robustness check. 19

Figure S18. Significant interactions between exposed population to wildfires and floods with income and residence area on climate policy support. 21

Table S1. Weighted linear multilevel regression predicting policy support for droughts (random intercepts across countries)... 22

Table S2. Weighted linear multilevel regression predicting policy support for heatwaves (random intercepts across countries) 23

Table S3. Weighted linear multilevel regression predicting policy support for heavy precipitation (random intercepts across countries) 24

Table S4. Weighted linear multilevel regression predicting policy support for river floods (random intercepts across countries) 25

Table S5. Weighted linear multilevel regression predicting policy support for wildfires (random intercepts across countries)... 26

Table S6. Weighted linear multilevel regression predicting policy support for tropical cyclones (random intercepts across countries) 27

Table S7. Weighted linear multilevel regression predicting policy support for European winter storms (random intercepts across countries)........ 28
Table S8. Weighted linear multilevel regression predicting mean level of subjective attribution across events (random intercepts across countries) 29
Table S9. Mean and standard deviation for extreme weather event variables. 30
Table S10. Reliability (omega) of subjective attribution scale across 67 countries in ascending order 31
Table S11. Reliability (omega) of aggregate policy support scale across 66 countries in ascending order 32
Table S12. Polychoric exploratory factor analysis (EFA) with items measuring climate policy support .. 33
Table S13. Definitions of extreme weather events and data sources. 34
Reference 36

Figure S1. Mean and standard errors of climate policy support (mean index), N = 67,626

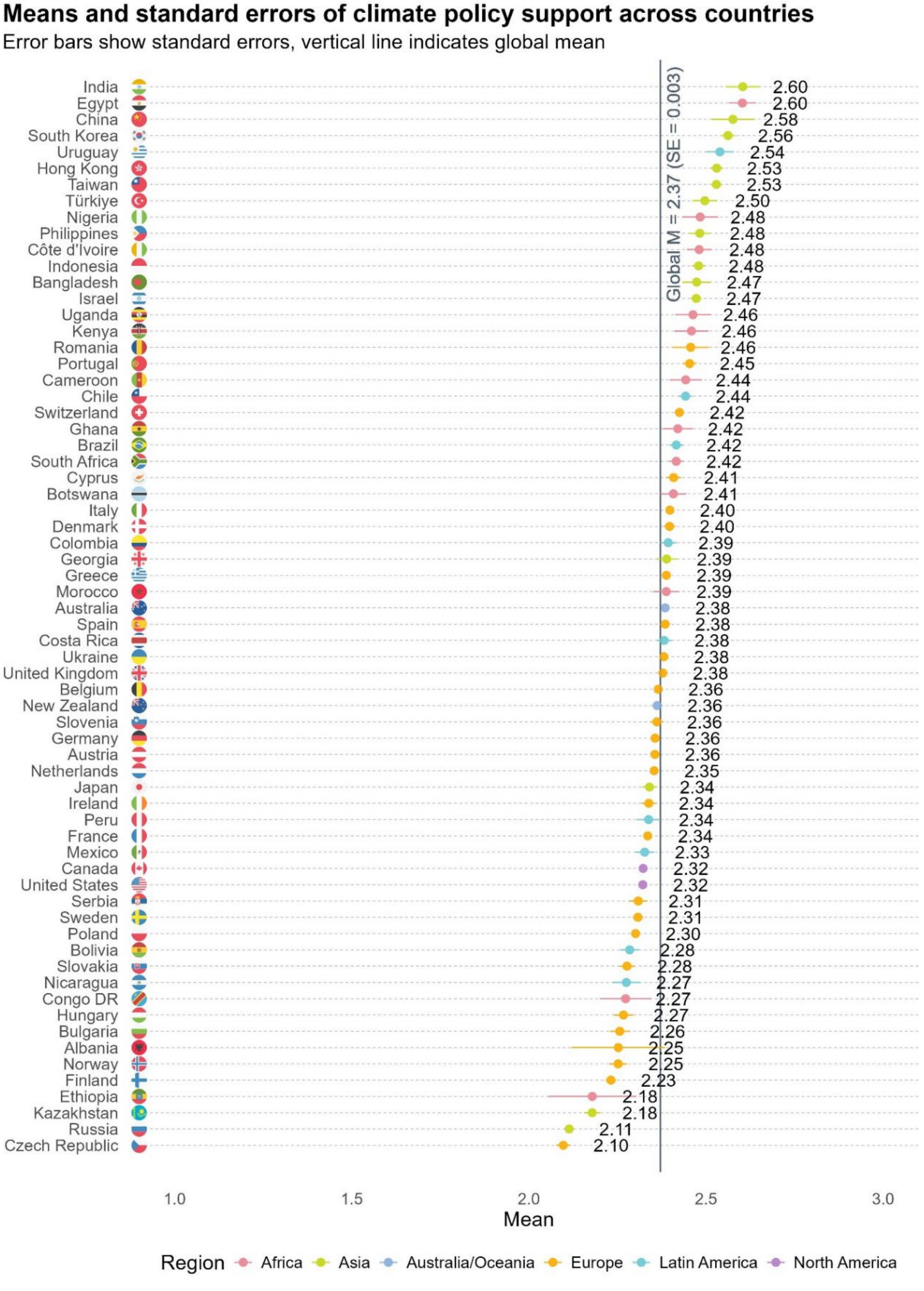

Note: Circular flags are plotted using the 'ggflags' package (Auguie et al., 2024) and are taken from EmojiOne (CC-BY-4.0/MIT): https://github.com/13rac1/emojione-color-font/blob/master/LICENSE.md

Figure S2. Mean and standard errors of support for taxes on carbon intense foods, $N = 64,038$

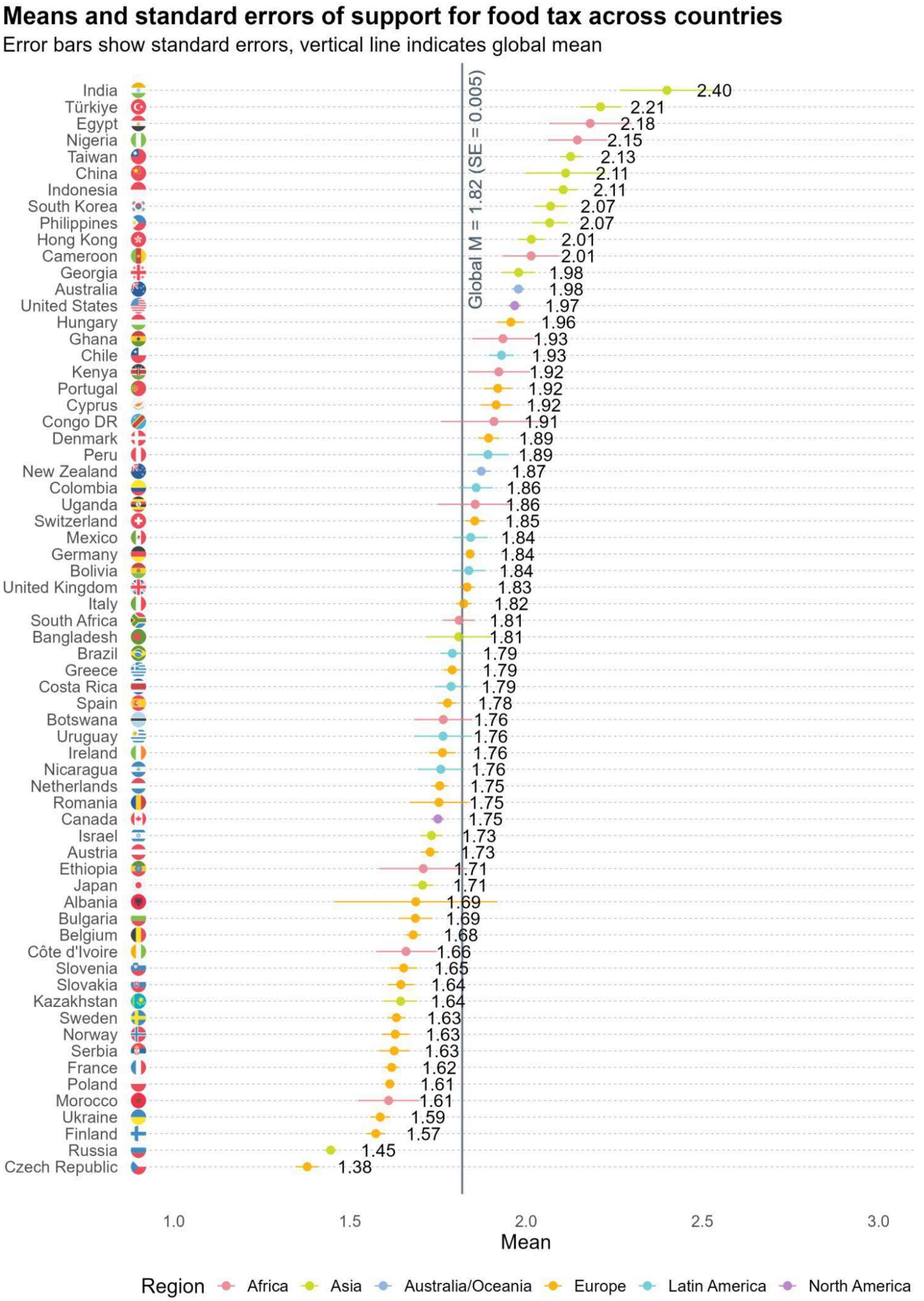

Note: Circular flags are plotted using the 'ggflags' package (Auguie et al., 2024) and are taken from EmojiOne (CC-BY-4.0/MIT): https://github.com/13rac1/emojione-color-font/blob/master/LICENSE.md

Figure S3. Mean and standard errors of support for fuel taxes, $N = 64{,}203$

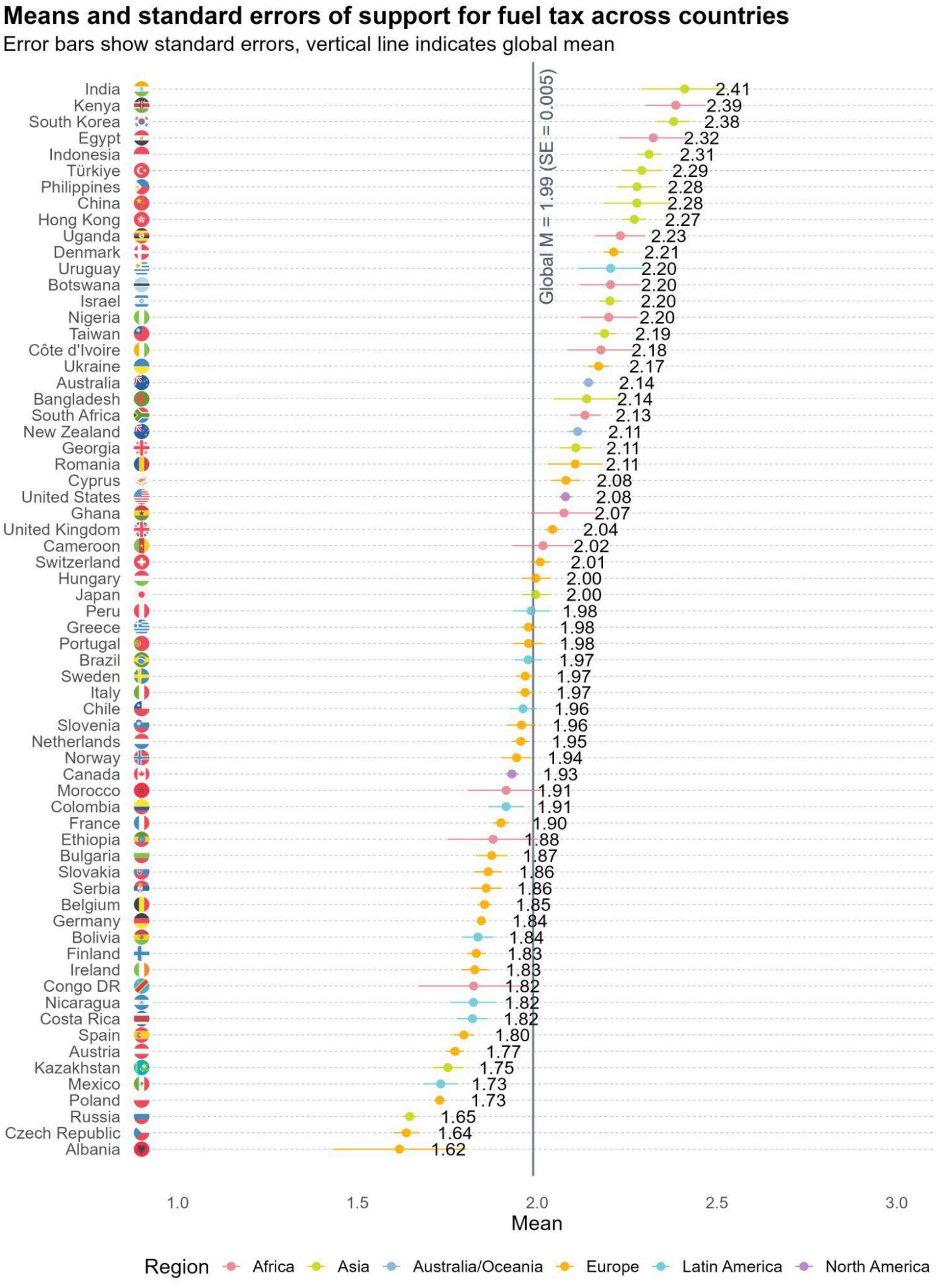

Note: Circular flags are plotted using the 'ggflags' package (Auguie et al., 2024) and are taken from EmojiOne (CC-BY-4.0/MIT): https://github.com/13rac1/emojione-color-font/blob/master/LICENSE.md

Figure S4. Mean and standard errors of support for public transport, *N* = 64,732

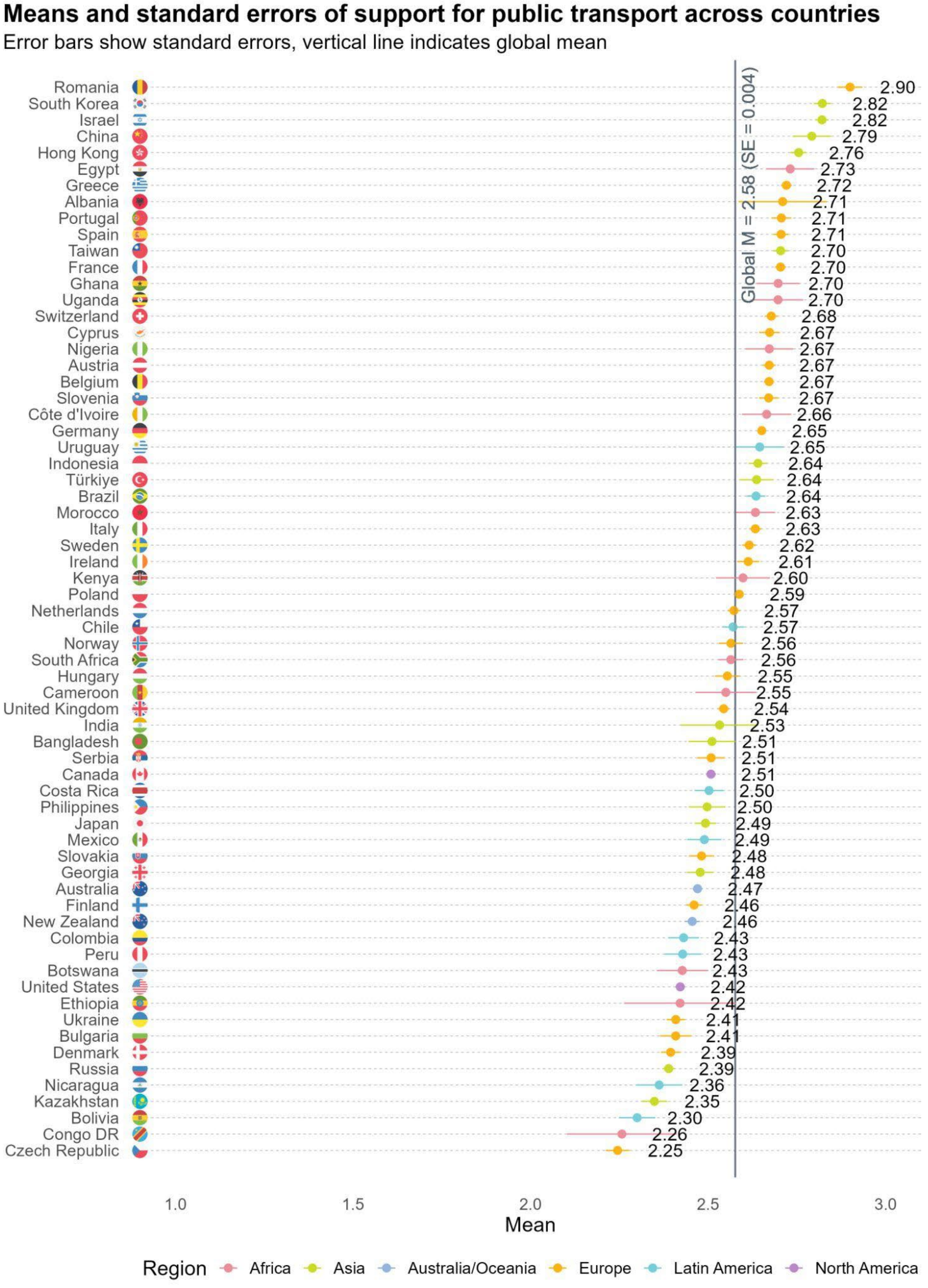

Note: Circular flags are plotted using the 'ggflags' package (Auguie et al., 2024) and are taken from EmojiOne (CC-BY-4.0/MIT): https://github.com/13rac1/emojione-color-font/blob/master/LICENSE.md

Figure S5. Mean and standard errors of support for sustainable energy, $N = 64{,}278$

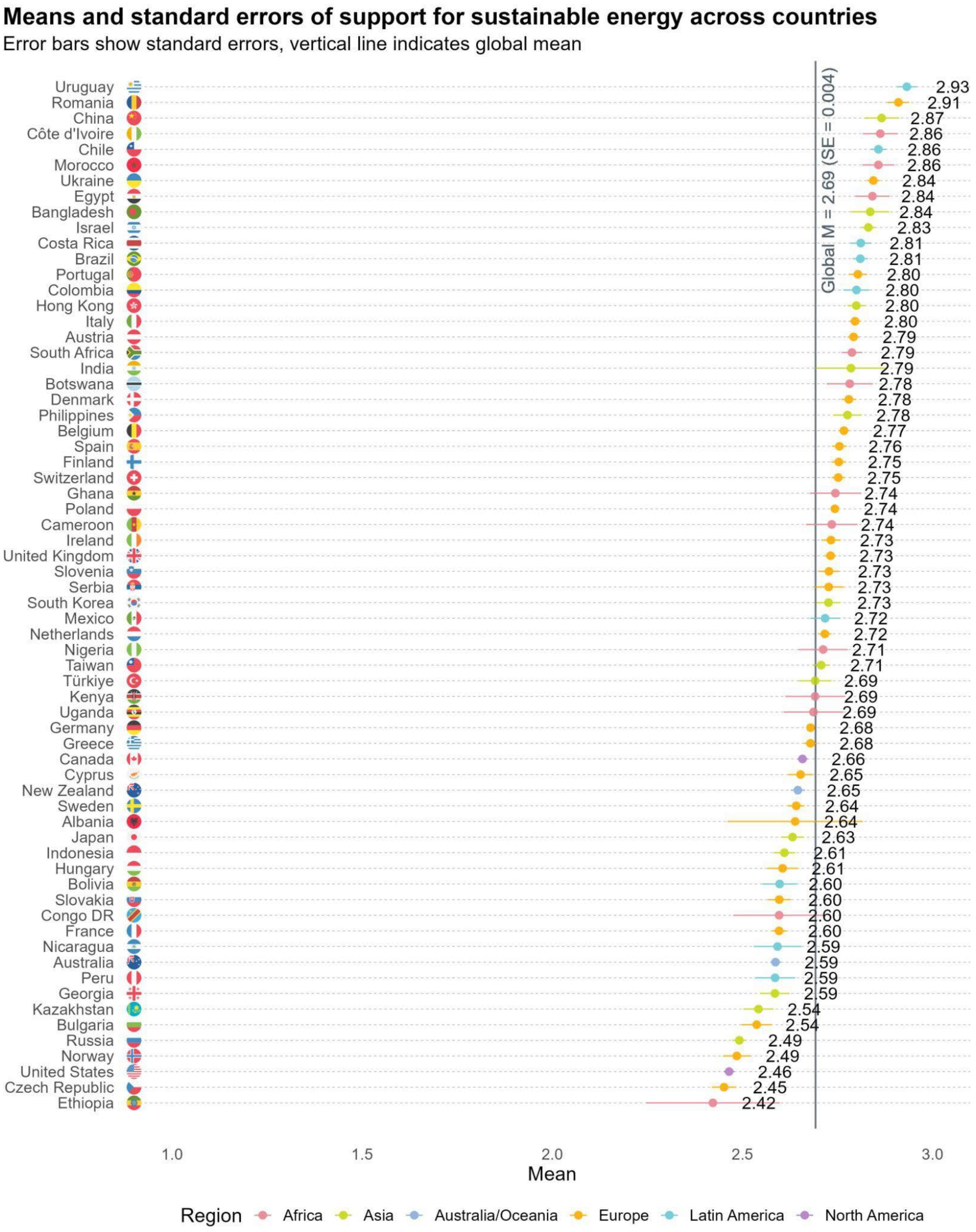

Note: Circular flags are plotted using the 'ggflags' package (Auguie et al., 2024) and are taken from EmojiOne (CC-BY-4.0/MIT): https://github.com/13rac1/emojione-color-font/blob/master/LICENSE.md

Figure S6. Mean and standard errors of support for protecting forested and land areas, N = 63,566

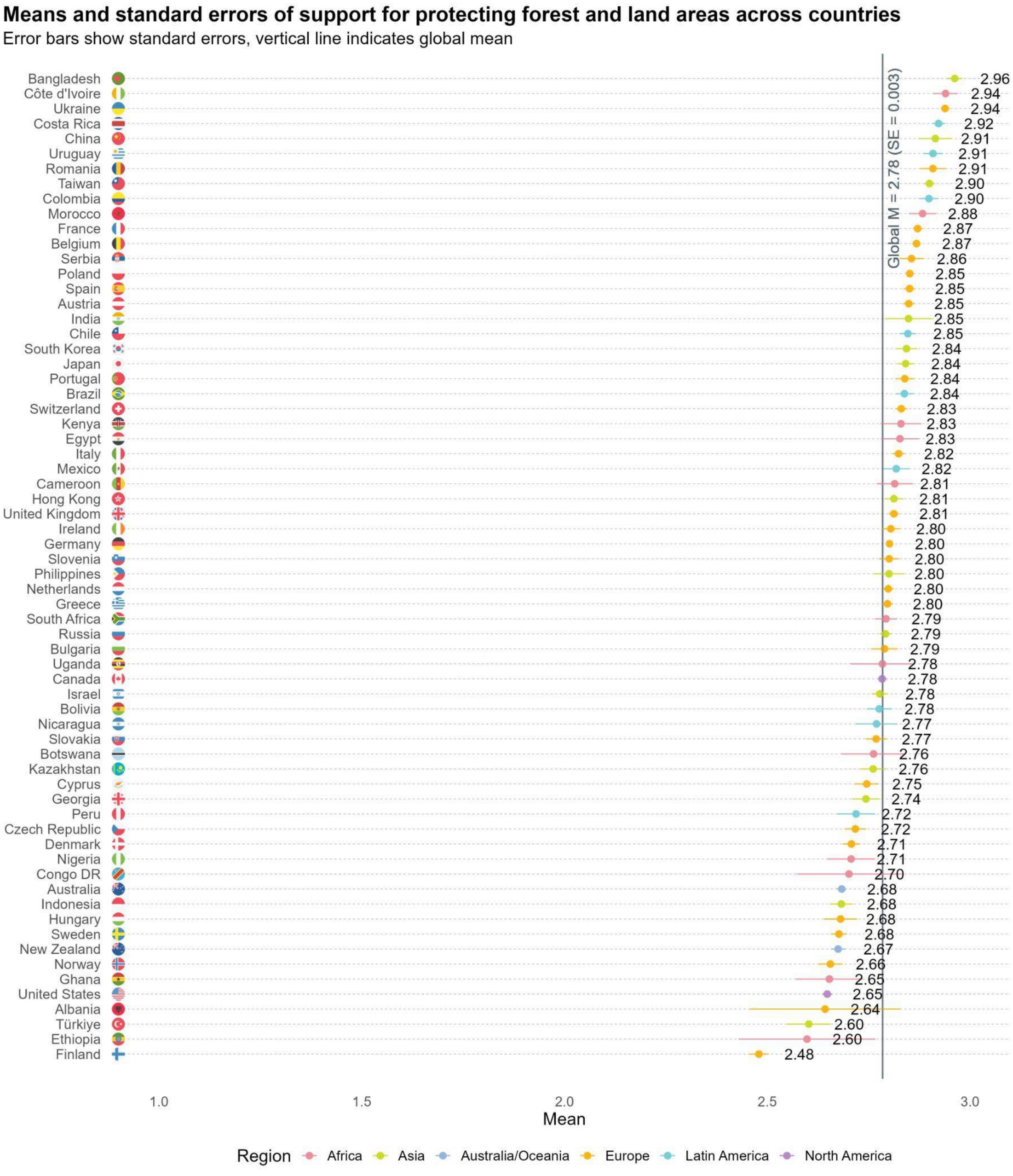

Note: Circular flags are plotted using the 'ggflags' package (Auguie et al., 2024) and are taken from EmojiOne (CC-BY-4.0/MIT): https://github.com/13rac1/emojione-color-font/blob/master/LICENSE.md

Figure S7. Weighted linear multilevel models comparing effects across policy measures (aggregate, taxes, green transition) for each extreme weather event (random intercepts across countries).

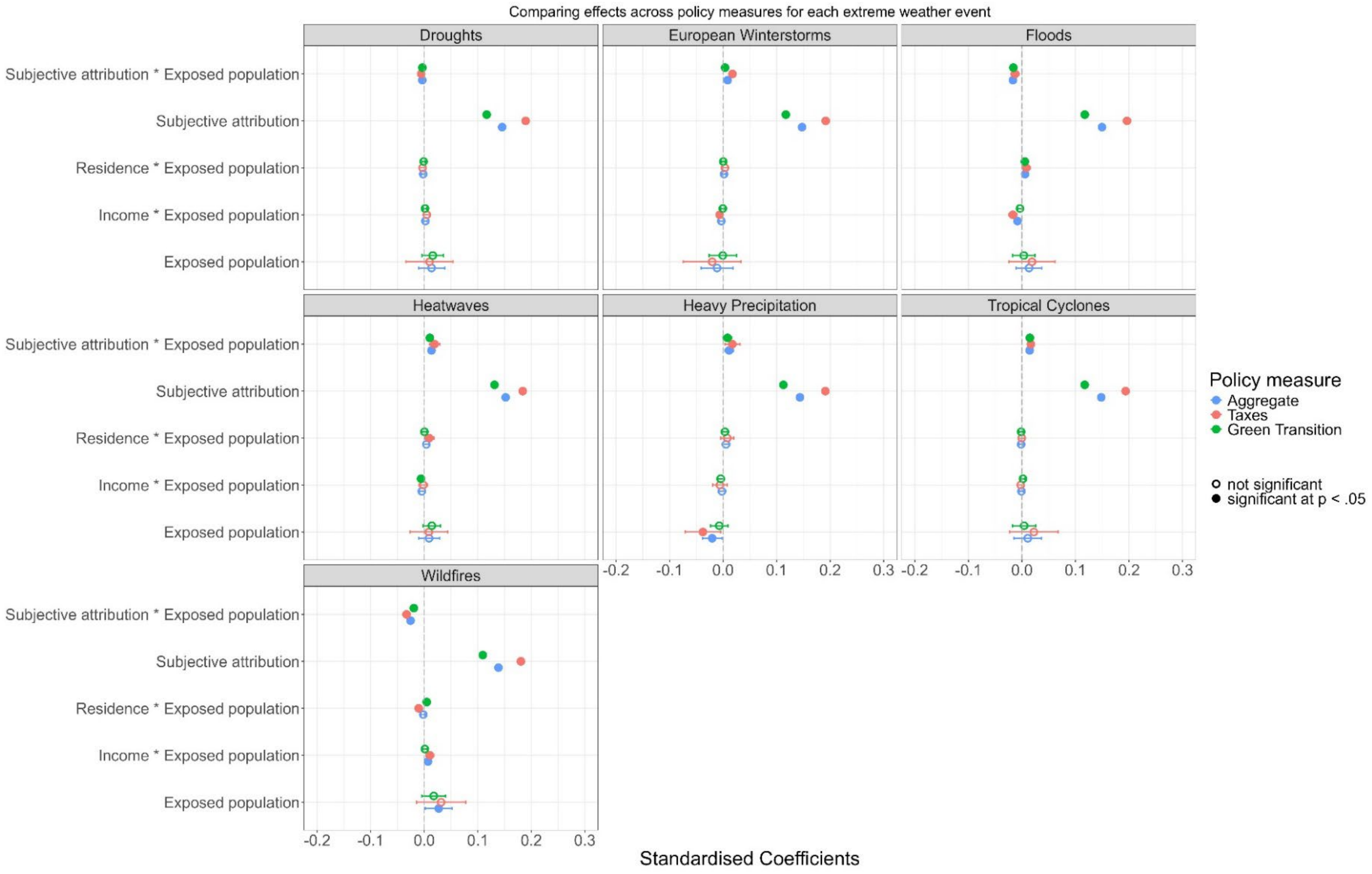

Note: These models include socio-demographic variables. Error bars denote 95% confidence intervals. Circles denote standardized estimates. Filled circles denote significant effects at $p < .05$.

Figure S8. Effect of male gender on climate policy support across countries.

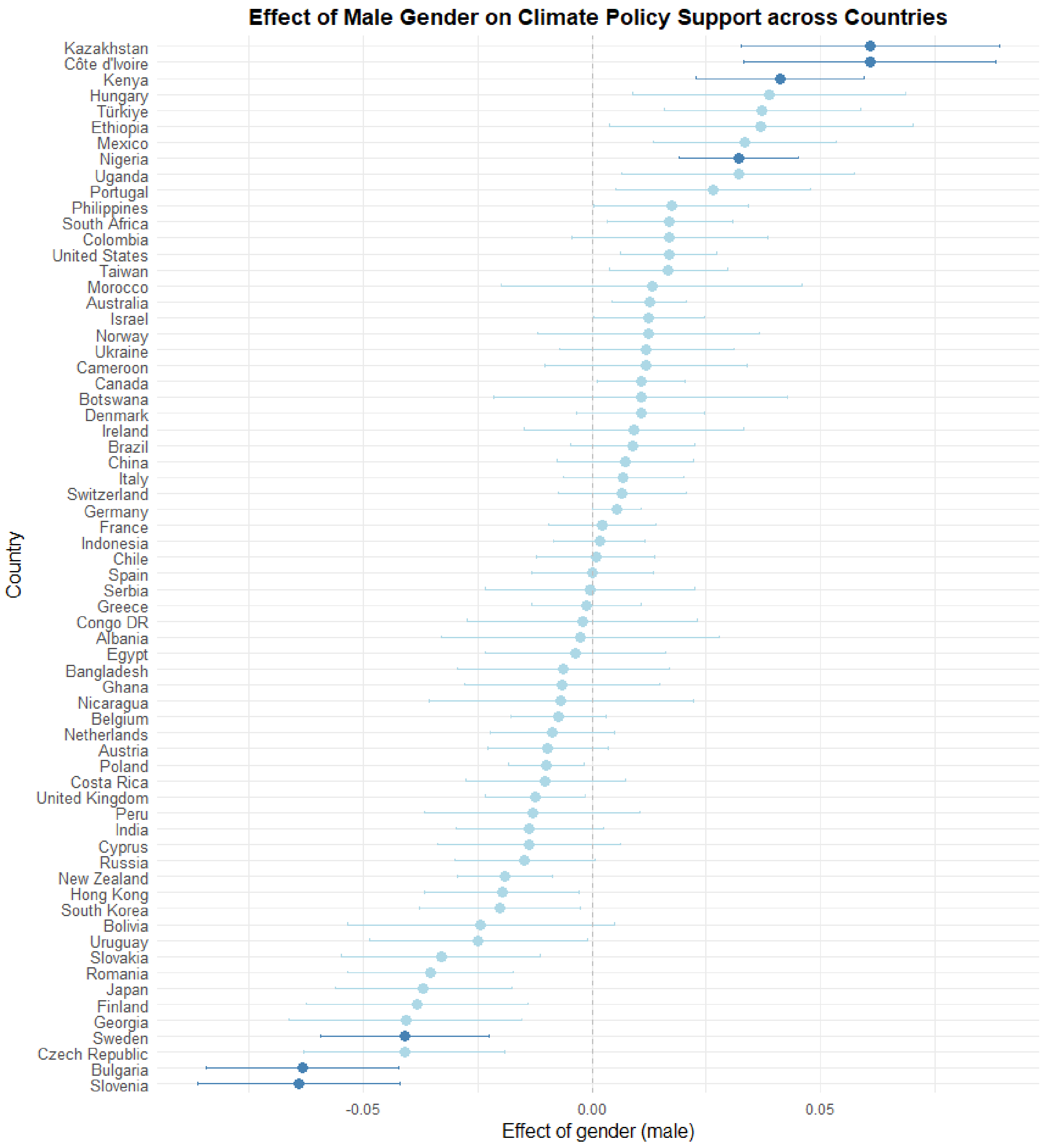

Note: The effects of gender on policy support were calculated with individual linear regressions for each country, with socio-demographic variables as independent variables. These models include data from 66 countries (excluding Malaysia and Argentina where policy support was not assessed). Significant effects at $p > .05$ are shown in dark blue. The number of participants in each country can be found in Mede et al. (2025).

Figure S9. Mean and standard errors of subjective attribution across countries, *N* = 69,163

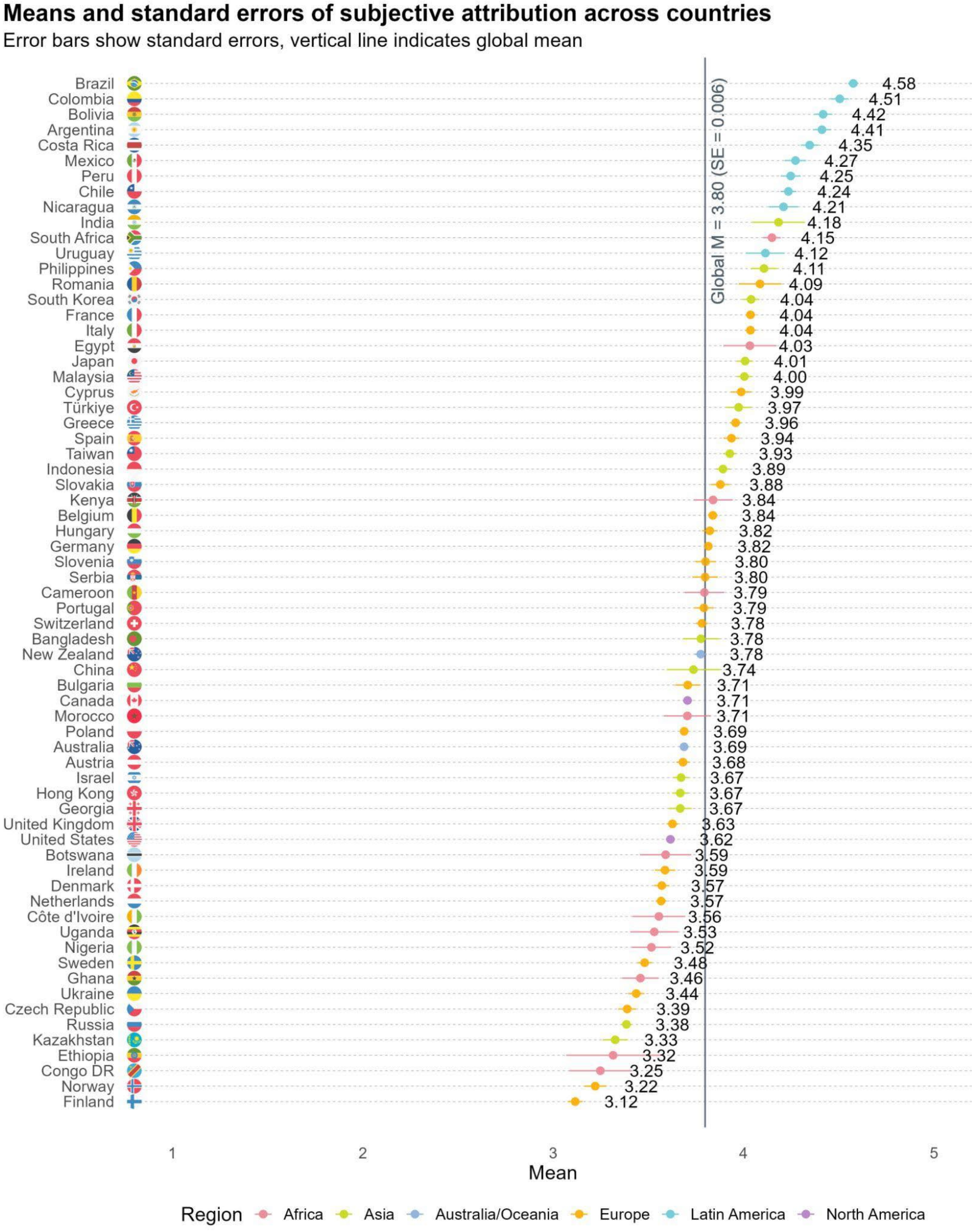

Note: Circular flags are plotted using the 'ggflags' package (Auguie et al., 2024) and are taken from EmojiOne (CC-BY-4.0/MIT): https://github.com/13rac1/emojione-color-font/blob/master/LICENSE.md

Figure S10. Random effects for subjective attribution (heatwaves) on policy support

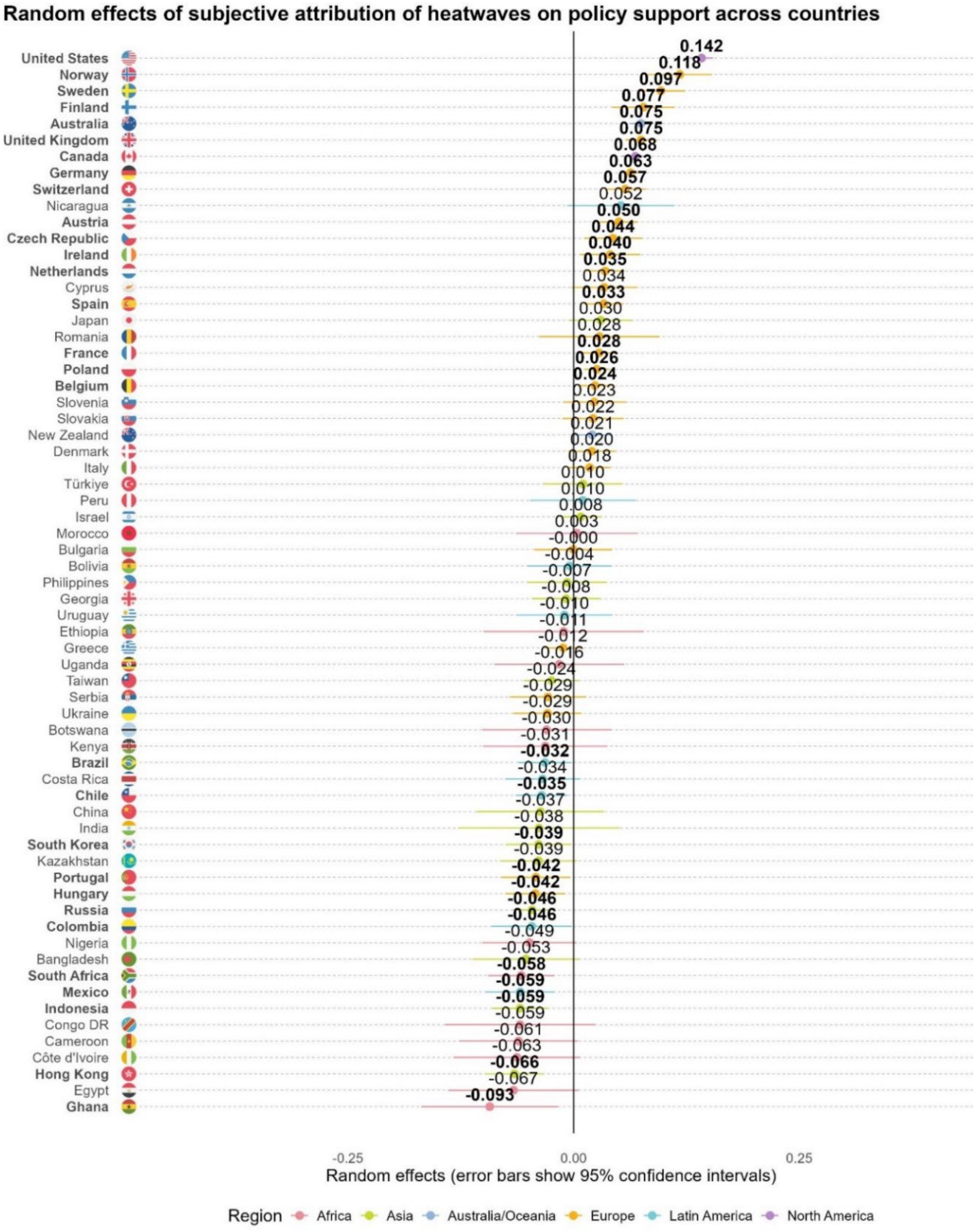

Note: Dots indicate point estimates of random effects, horizontal lines indicate 95% confidence intervals based on two-sided t tests. Effects significant at $p < .05$ are printed in bold. Total $N = 46{,}964$. Circular flags are plotted using the 'ggflags' package (Auguie et al., 2024) and are taken from EmojiOne (CC-BY-4.0/MIT): https://github.com/13rac1/emojione-color-font/blob/master/LICENSE.md

Figure S11. Random effects for subjective attribution (droughts) on policy support

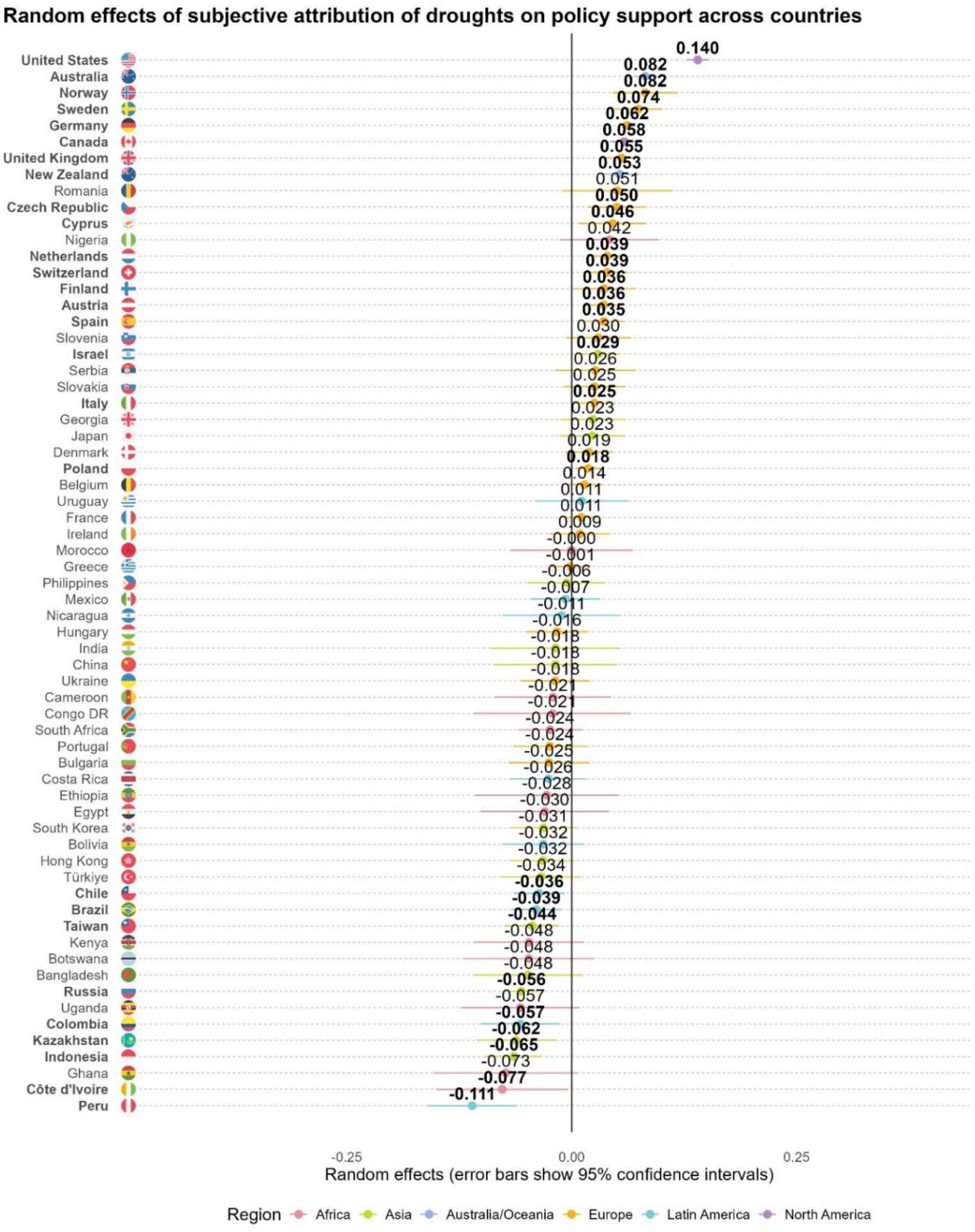

Note: Dots indicate point estimates of random effects, horizontal lines indicate 95% confidence intervals based on two-sided t tests. Effects significant at $p < .05$ are printed in bold. Total $N = 46{,}947$. Circular flags are plotted using the 'ggflags' package (Auguie et al., 2024) and are taken from EmojiOne (CC-BY-4.0/MIT): https://github.com/13rac1/emojione-color-font/blob/master/LICENSE.md

Figure S12. Random effects for subjective attribution (river floods) on policy support

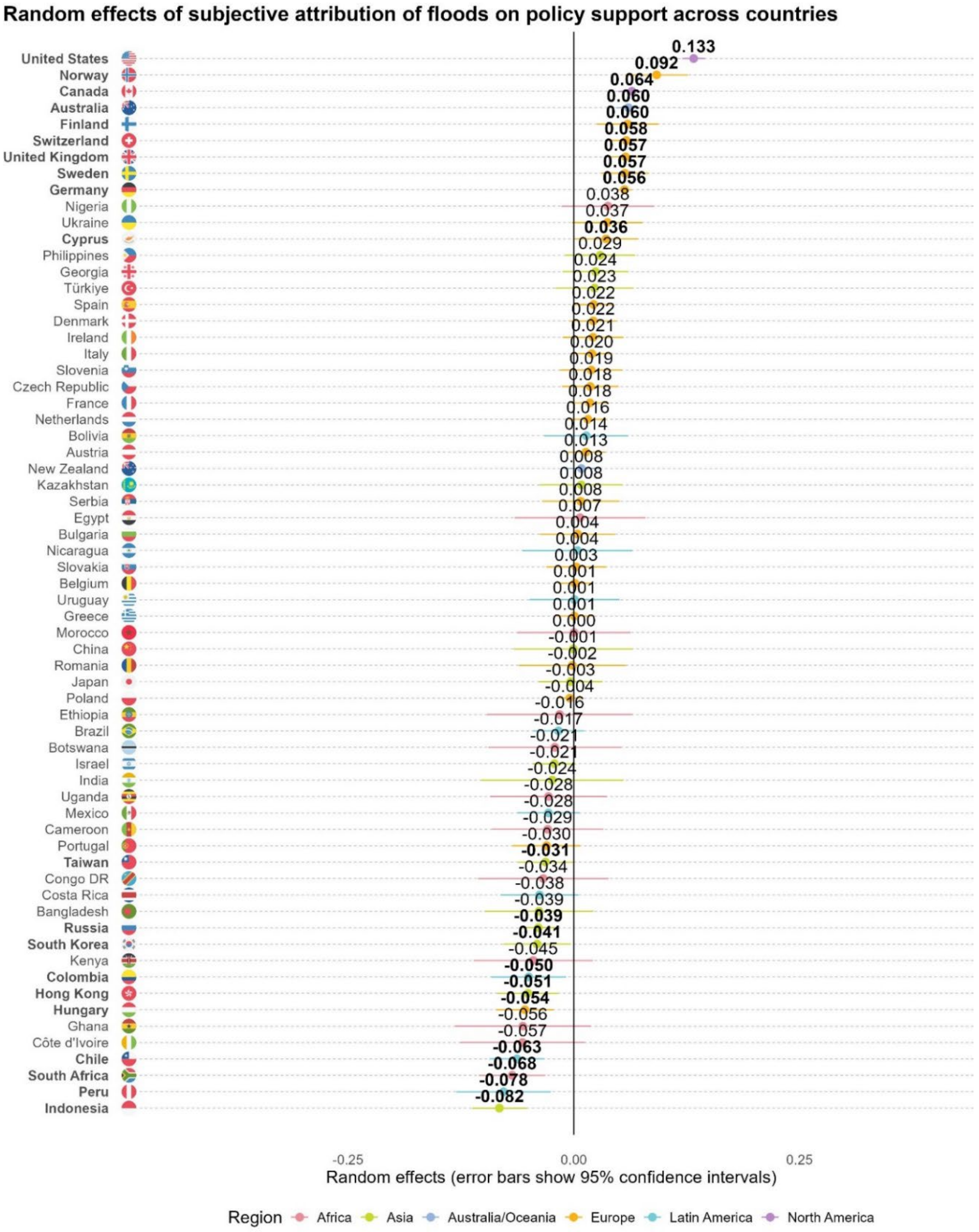

Note: Dots indicate point estimates of random effects, horizontal lines indicate 95% confidence intervals based on two-sided t tests. Effects significant at $p < .05$ are printed in bold. Total $N = 46{,}964$. Circular flags are plotted using the 'ggflags' package (Auguie et al., 2024) and are taken from EmojiOne (CC-BY-4.0/MIT): https://github.com/13rac1/emojione-color-font/blob/master/LICENSE.md

Figure S13. Random effects for subjective attribution (wildfires) on policy support

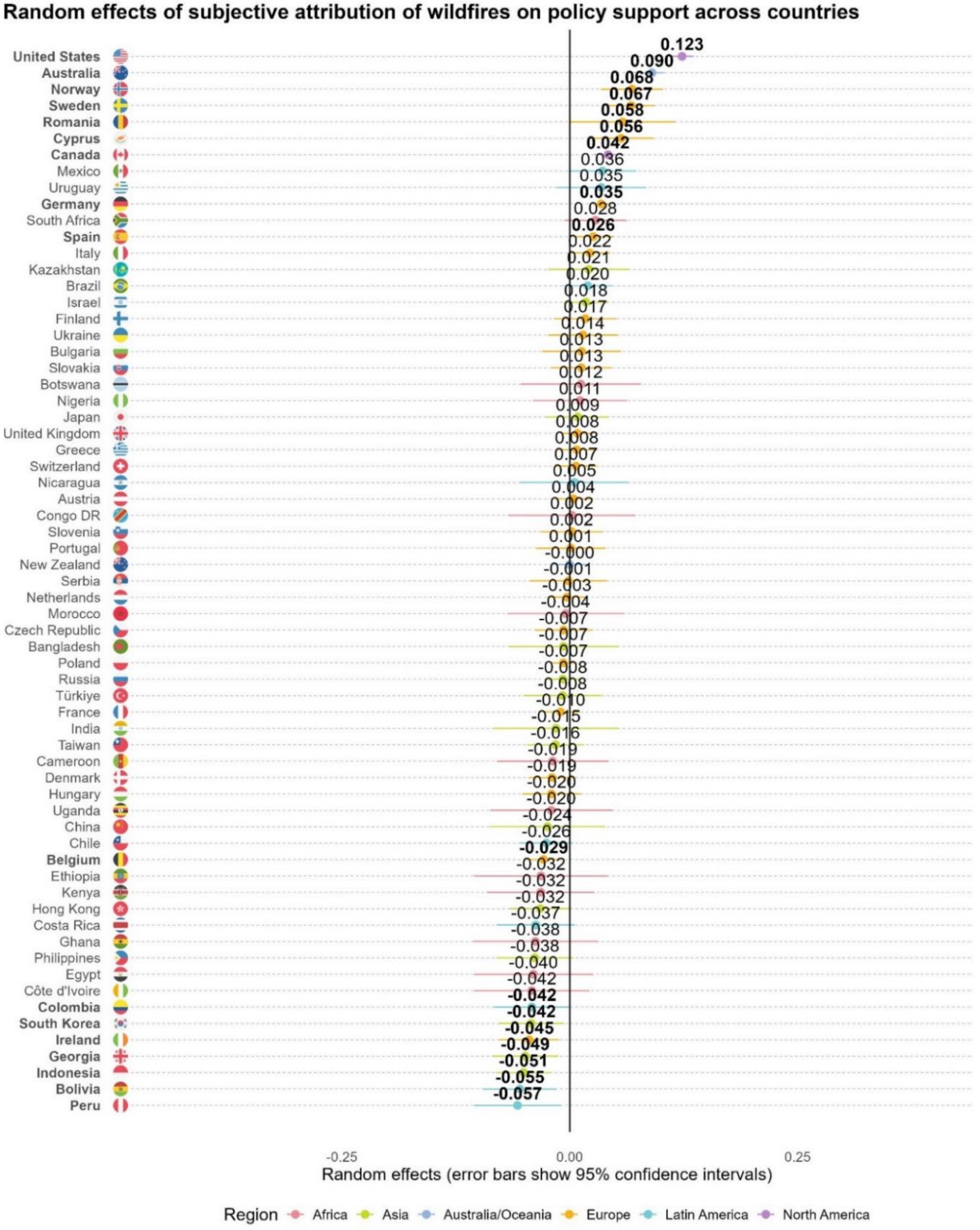

Note: Dots indicate point estimates of random effects, horizontal lines indicate 95% confidence intervals based on two-sided t tests. Effects significant at $p < .05$ are printed in bold. Total $N = 46,954$. Circular flags are plotted using the ‘ggflags’ package (Auguie et al., 2024) and are taken from EmojiOne (CC-BY-4.0/MIT): https://github.com/13rac1/emojione-color-font/blob/master/LICENSE.md

Figure S14. Random effects for subjective attribution (tropical cyclones) on policy support

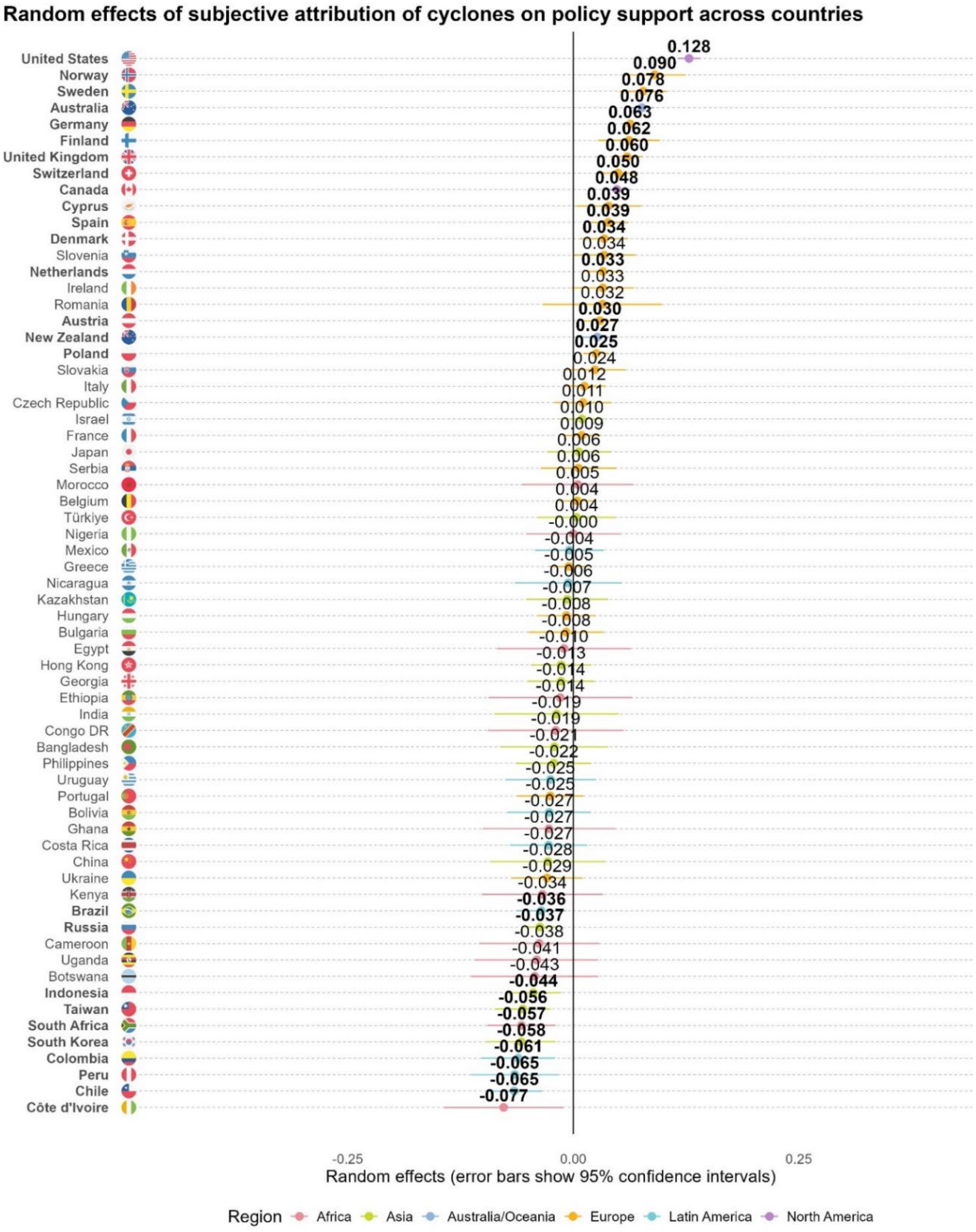

Note: Dots indicate point estimates of random effects, horizontal lines indicate 95% confidence intervals based on two-sided t tests. Effects significant at $p < .05$ are printed in bold. Total $N = 46{,}961$. Circular flags are plotted using the 'ggflags' package (Auguie et al., 2024) and are taken from EmojiOne (CC-BY-4.0/MIT): https://github.com/13rac1/emojione-color-font/blob/master/LICENSE.md

Figure S15. Random effects for subjective attribution (heavy precipitation) on policy support

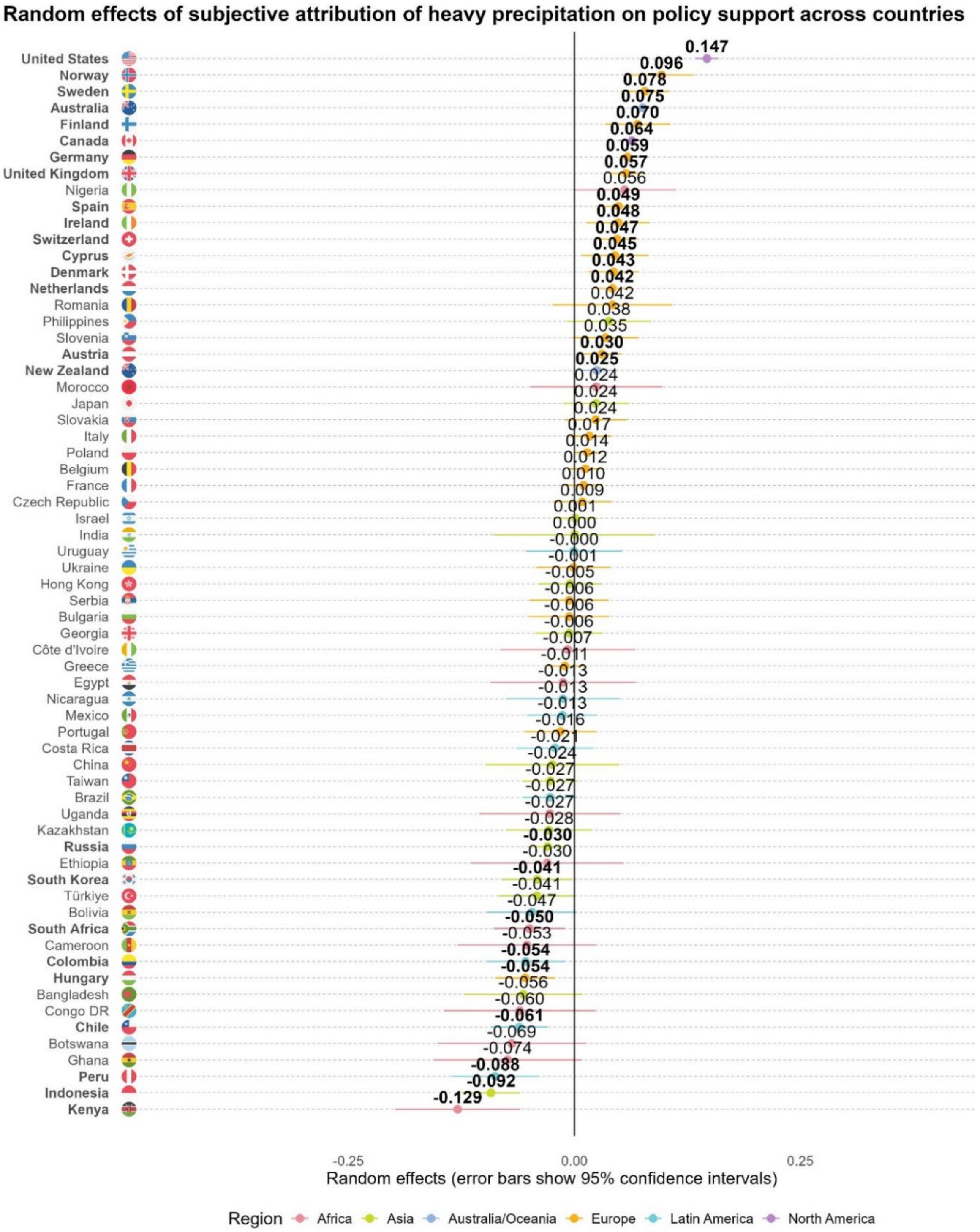

Note: Dots indicate point estimates of random effects, horizontal lines indicate 95% confidence intervals based on two-sided t tests. Effects significant at $p < .05$ are printed in bold. Total $N = 46{,}958$. Circular flags are plotted using the 'ggflags' package (Auguie et al., 2024) and are taken from EmojiOne (CC-BY-4.0/MIT): https://github.com/13rac1/emojione-color-font/blob/master/LICENSE.md

Figure S16. Random effects for subjective attribution (European winterstorms) on policy support

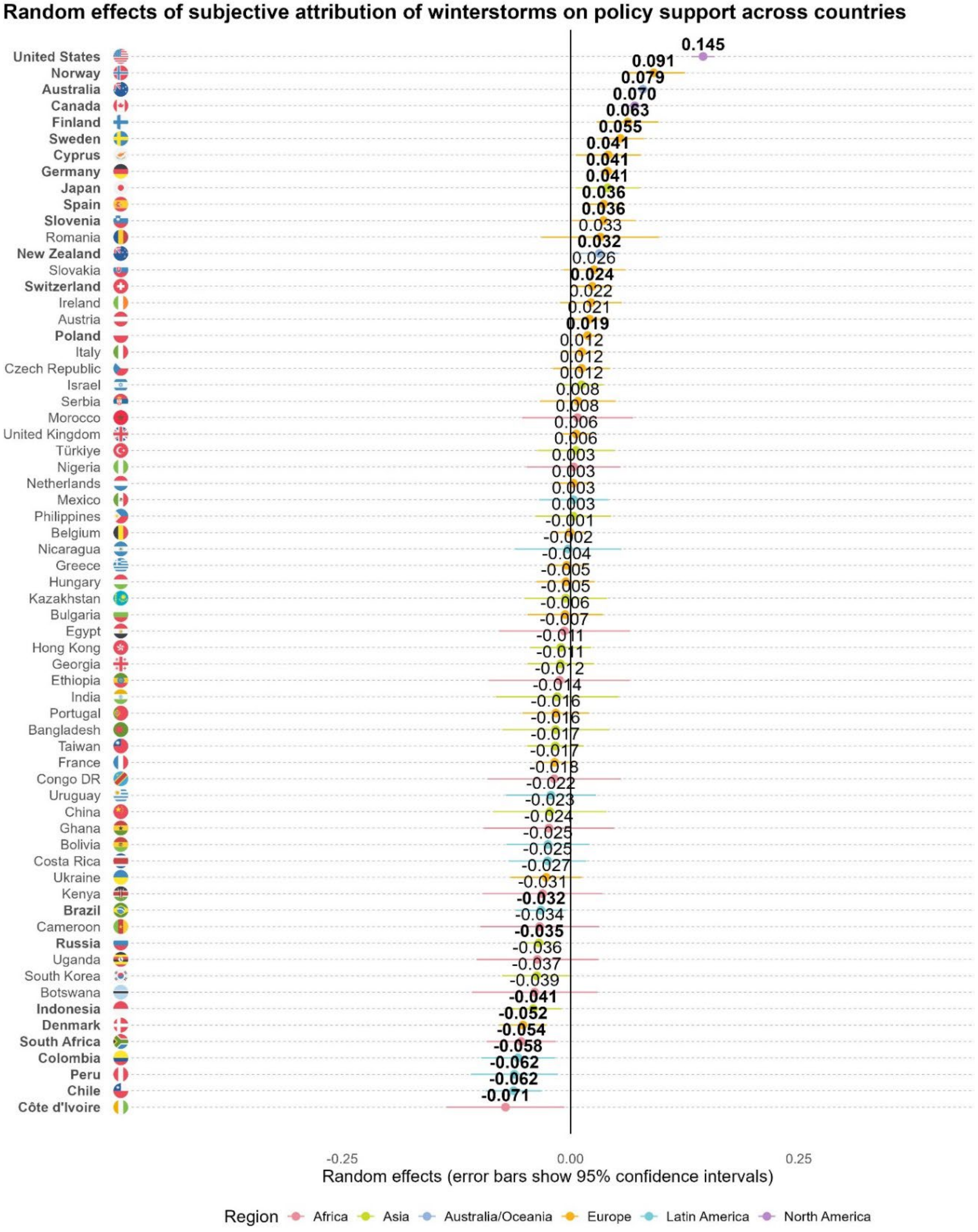

Note: Dots indicate point estimates of random effects, horizontal lines indicate 95% confidence intervals based on two-sided t tests. Effects significant at $p < .05$ are printed in bold. Total $N = 46{,}961$. Circular flags are plotted using the 'ggflags' package (Auguie et al., 2024) and are taken from EmojiOne (CC-BY-4.0/MIT): https://github.com/13rac1/emojione-color-font/blob/master/LICENSE.md

Figure S17. Comparison of three models as robustness check.

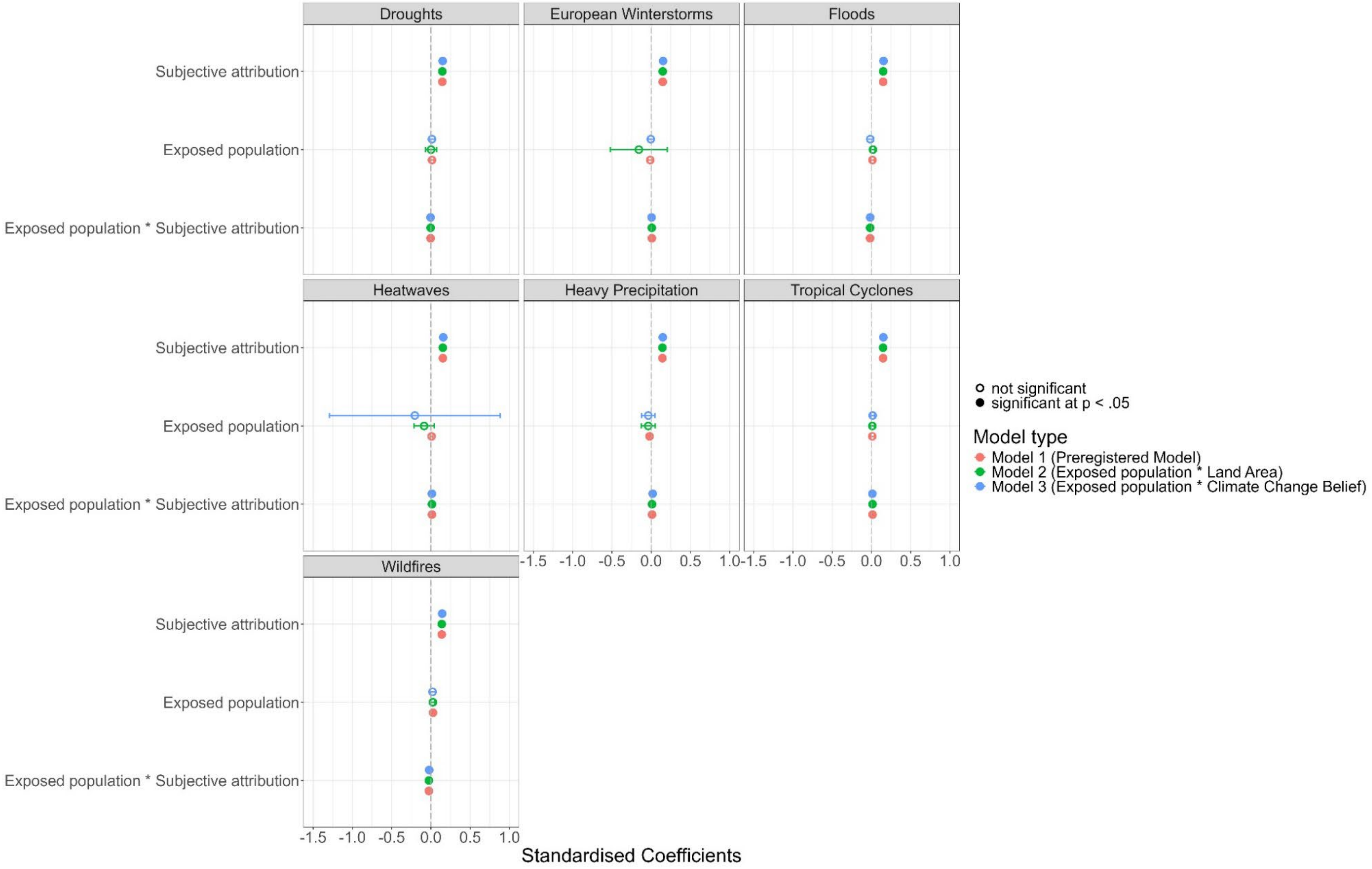

Note: Model 1 is the preregistered multilevel model shown in Figure 4 (data is available for 65 countries). Model 2 additionally includes an interaction effect for exposed population and land area. Model 3 additionally includes an interaction effect for exposed population and climate change belief on the country level (data was available for 48 countries). All models include random intercepts across countries and control for socio-demographic variables and two additional interaction terms (exposed population × income and exposed population × residence area). Error bars denote 95% confidence intervals. Circles denote standardized estimates. Filled circles denote significant effects at $p < .05$.

Figure S18. Significant interactions between exposed population to wildfires and floods with income and residence area on climate policy support.

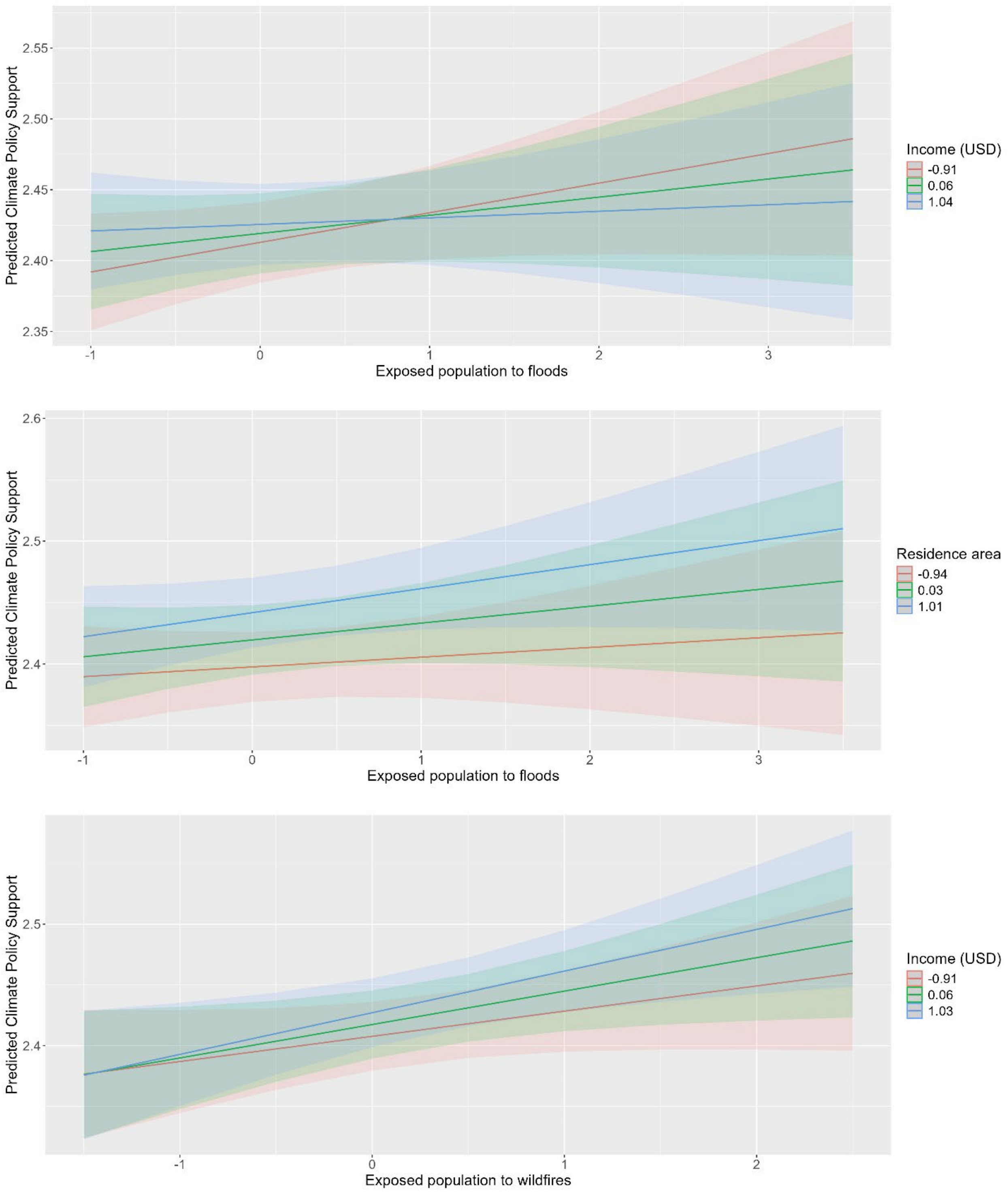

Table S1. Weighted linear multilevel regression predicting policy support for droughts (random intercepts across countries)

	**Step 1: Exposed population**				**Step 2: Moderation analyses**			
Predictors	*Beta*	*SE*	*Statistic*	*p*	*Beta*	*SE*	*Statistic*	*p*
Intercept	2.444	0.015	167.806	**<.001**	2.443	0.015	166.616	**<.001**
Gender (male)	0.000	0.002	-0.144	.886	0.008	0.002	4.248	**<.001**
Age	-0.015	0.002	-8.635	**<.001**	-0.019	0.002	-11.291	**<.001**
Education (tertiary)	0.038	0.002	19.246	**<.001**	0.032	0.002	17.015	**<.001**
Income	0.0100	0.002	5.125	**<.001**	0.008	0.002	4.528	**<.001**
Residence place (urban)	0.027	0.002	14.238	**<.001**	0.024	0.002	13.269	**<.001**
Political orientation (right)	-0.056	0.002	-24.873	**<.001**	-0.039	0.002	-18.258	**<.001**
Political orientation (conservative)	-0.042	0.002	-18.514	**<.001**	-0.032	0.002	-14.82	**<.001**
Religiosity	0.024	0.002	12.011	**<.001**	0.018	0.002	9.769	**<.001**
Exposed population to droughts	0.014	0.012	1.11	.272	0.014	0.012	1.101	.275
Subjective attribution					0.146	0.002	80.37	**<.001**
Exposed population to droughts * Subjective attribution					-0.004	0.002	-2.185	**.029**
Exposed population to droughts * Income					0.002	0.002	1.349	.177
Exposed population to droughts * Residence (urban)					-0.002	0.002	-1.119	.263
**Random Effects**								
σ^2	0.1				0.09			
τ_{00}	0.01				0.01			
ICC	0.11				0.12			
N	66 Countries				65 Countries			
Observations	47197				46947			
Marginal R^2 / Conditional R^2	0.079 / 0.181				0.222 / 0.319			
AIC	78059.397				70978.492			

Table S2. Weighted linear multilevel regression predicting policy support for heatwaves (random intercepts across countries)

	**Step 1: Exposed population**				**Step 2: Moderation analyses**			
Predictors	*Beta*	*SE*	*Statistic*	*p*	*Beta*	*SE*	*Statistic*	*p*
Intercept	2.448	0.015	167.397	**<.001**	2.446	0.015	166.278	**<.001**
Gender (male)	0	0.002	-0.147	.883	0.009	0.002	5.251	**<.001**
Age	-0.015	0.002	-8.646	**<.001**	-0.013	0.002	-7.966	**<.001**
Education (tertiary)	0.038	0.002	19.238	**<.001**	0.032	0.002	17.512	**<.001**
Income	0.01	0.002	5.127	**<.001**	0.008	0.002	4.399	**<.001**
Residence place (urban)	0.027	0.002	14.24	**<.001**	0.022	0.002	12.516	**<.001**
Political orientation (right)	-0.056	0.002	-24.872	**<.001**	-0.036	0.002	-17.223	**<.001**
Political orientation (conservative)	-0.042	0.002	-18.514	**<.001**	-0.028	0.002	-13.389	**<.001**
Religiosity	0.024	0.002	12.009	**<.001**	0.019	0.002	10.164	**<.001**
Exposed population to heatwaves	0.008	0.01	0.844	.402	0.009	0.01	0.905	.369
Subjective attribution					0.152	0.002	83.896	**<.001**
Exposed population to heatwaves * Subjective attribution					0.014	0.003	5.079	**<.001**
Exposed population to heatwaves * Income					-0.005	0.002	-1.909	.056
Exposed population to heatwaves * Residence (urban)					0.004	0.002	1.773	.076
**Random Effects**								
σ^2	0.1				0.09			
τ_{00}	0.01				0.01			
ICC	0.11				0.13			
N	66 Countries				65 Countries			
Observations	47197				46964			
Marginal R^2 / Conditional R^2	0.078 / 0.181				0.235 / 0.333			
AIC	78060.363				70337.589			

Table S3. Weighted linear multilevel regression predicting policy support for heavy precipitation (random intercepts across countries)

	Step 1: Exposed population				Step 2: Moderation analyses			
Predictors	*Beta*	*SE*	*Statistic*	*p*	*Beta*	*SE*	*Statistic*	*p*
Intercept	2.444	0.014	172.426	**<.001**	2.44	0.014	177.269	**<.001**
Gender (male)	0	0.002	-0.142	.887	0.009	0.002	4.997	**<.001**
Age	-0.015	0.002	-8.645	**<.001**	-0.022	0.002	-12.948	**<.001**
Education (tertiary)	0.038	0.002	19.248	**<.001**	0.033	0.002	17.821	**<.001**
Income	0.01	0.002	5.118	**<.001**	0.009	0.002	4.634	**<.001**
Residence place (urban)	0.027	0.002	14.232	**<.001**	0.023	0.002	12.868	**<.001**
Political orientation (right)	-0.056	0.002	-24.89	**<.001**	-0.04	0.002	-18.819	**<.001**
Political orientation (conservative)	-0.042	0.002	-18.493	**<.001**	-0.031	0.002	-14.589	**<.001**
Religiosity	0.024	0.002	11.998	**<.001**	0.016	0.002	8.767	**<.001**
Exposed population to heavy precipitation	-0.02	0.01	-2.12	**.037**	-0.02	0.009	-2.202	**.031**
Subjective attribution					0.144	0.002	77.703	**<.001**
Exposed population to heavy precipitation * Subjective attribution					0.011	0.004	2.764	**.006**
Exposed population to heavy precipitation * Income					-0.002	0.004	-0.58	.562
Exposed population to heavy precipitation * Residence (urban)					0.005	0.004	1.509	.131
**Random Effects**								
σ^2	0.1				0.09			
τ_{00}	0.01				0.01			
ICC	0.11				0.11			
N	66 Countries				65 Countries			
Observations	47197				46958			
Marginal R^2 / Conditional R^2	0.082 / 0.178				0.225 / 0.312			
AIC	78056.738				71091.978			

Table S4. Weighted linear multilevel regression predicting policy support for river floods (random intercepts across countries)

	**Step 1: Exposed population**				**Step 2: Moderation analyses**			
Predictors	*Beta*	*SE*	*Statistic*	*p*	*Beta*	*SE*	*Statistic*	*p*
Intercept	2.442	0.015	164.112	**<.001**	2.437	0.014	168.581	**<.001**
Gender (male)	0.000	0.002	-0.146	.884	0.012	0.002	6.727	**<.001**
Age	-0.015	0.002	-8.632	**<.001**	-0.023	0.002	-14.102	**<.001**
Education (tertiary)	0.038	0.002	19.272	**<.001**	0.032	0.002	17.414	**<.001**
Income	0.01	0.002	5.124	**<.001**	0.006	0.002	3.472	**.001**
Residence place (urban)	0.027	0.002	14.24	**<.001**	0.023	0.002	12.471	**<.001**
Political orientation (right)	-0.056	0.002	-24.875	**<.001**	-0.038	0.002	-18.201	**<.001**
Political orientation (conservative)	-0.042	0.002	-18.51	**<.001**	-0.029	0.002	-13.792	**<.001**
Religiosity	0.024	0.002	12.005	**<.001**	0.016	0.002	8.657	**<.001**
Exposed population to floods	0.016	0.013	1.275	.207	0.013	0.012	1.094	.278
Subjective attribution					0.15	0.002	79.847	**<.001**
Exposed population to floods * Subjective attribution					-0.017	0.002	-7.632	**<.001**
Exposed population to floods * Income					-0.008	0.002	-3.971	**<.001**
Exposed population to floods * Residence (urban)					0.006	0.002	2.801	**.005**
**Random Effects**								
σ^2	0.1				0.09			
τ_{00}	0.01				0.01			
ICC	0.11				0.12			
N	66 Countries				65 Countries			
Observations	47197				46964			
Marginal R^2 / Conditional R^2	0.080 / 0.181				0.236 / 0.327			
AIC	78058.988				70239.349			

Table S5. Weighted linear multilevel regression predicting policy support for wildfires (random intercepts across countries)

	Step 1: Exposed population				Step 2: Moderation analyses			
Predictors	*Beta*	*SE*	*Statistic*	*p*	*Beta*	*SE*	*Statistic*	*p*
Intercept	2.439	0.015	166.725	**<.001**	2.435	0.014	169.736	**<.001**
Gender (male)	0.000	0.002	-0.137	.891	0.009	0.002	4.935	**<.001**
Age	-0.015	0.002	-8.619	**<.001**	-0.017	0.002	-9.931	**<.001**
Education (tertiary)	0.038	0.002	19.293	**<.001**	0.034	0.002	18.098	**<.001**
Income	0.01	0.002	5.122	**<.001**	0.01	0.002	5.254	**<.001**
Residence place (urban)	0.027	0.002	14.235	**<.001**	0.021	0.002	11.66	**<.001**
Political orientation (right)	-0.056	0.002	-24.871	**<.001**	-0.039	0.002	-18.414	**<.001**
Political orientation (conservative)	-0.042	0.002	-18.514	**<.001**	-0.031	0.002	-14.319	**<.001**
Religiosity	0.024	0.002	12.011	**<.001**	0.018	0.002	9.593	**<.001**
Exposed population to wildfires	0.027	0.013	2.002	**.049**	0.027	0.013	2.085	**.041**
Subjective attribution					0.139	0.002	73.62	**<.001**
Exposed population to wildfires * Subjective attribution					-0.026	0.002	-12.265	**<.001**
Exposed population to wildfires * Income					0.007	0.002	3.342	**.001**
Exposed population to wildfires * Residence (urban)					-0.002	0.002	-0.839	.402
**Random Effects**								
σ^2	0.1				0.09			
τ_{00}	0.01				0.01			
ICC	0.11				0.11			
N	66 Countries				65 Countries			
Observations	47197				46954			
Marginal R^2 / Conditional R^2	0.083 / 0.181				0.221 / 0.311			
AIC	78056.549				70915.9			

Table S6. Weighted linear multilevel regression predicting policy support for tropical cyclones (random intercepts across countries)

	**Step 1: Exposed population**				**Step 2: Moderation analyses**			
Predictors	*Beta*	*SE*	*Statistic*	*p*	*Beta*	*SE*	*Statistic*	*p*
Intercept	2.446	0.015	168.273	**<.001**	2.442	0.014	171.197	**<.001**
Gender (male)	0.000	0.002	-0.148	.882	0.01	0.002	5.54	**<.001**
Age	-0.015	0.002	-8.641	**<.001**	-0.022	0.002	-13.016	**<.001**
Education (tertiary)	0.038	0.002	19.237	**<.001**	0.033	0.002	17.884	**<.001**
Income	0.01	0.002	5.125	**<.001**	0.007	0.002	3.913	**<.001**
Residence place (urban)	0.027	0.002	14.238	**<.001**	0.022	0.002	12.715	**<.001**
Political orientation (right)	-0.056	0.002	-24.872	**<.001**	-0.039	0.002	-18.407	**<.001**
Political orientation (conservative)	-0.042	0.002	-18.514	**<.001**	-0.03	0.002	-14.138	**<.001**
Religiosity	0.024	0.002	12.01	**<.001**	0.016	0.002	8.865	**<.001**
Exposed population to cyclones	0.011	0.013	0.823	.414	0.011	0.013	0.832	.409
Subjective attribution					0.148	0.002	81.581	**<.001**
Exposed population to cyclones * Subjective attribution					0.014	0.002	7.615	**<.001**
Exposed population to cyclones * Income					-0.001	0.002	-0.46	.645
Exposed population to cyclones * Residence (urban)					-0.001	0.002	-0.796	.426
**Random Effects**								
σ^2	0.1				0.09			
τ_{00}	0.01				0.01			
ICC	0.11				0.12			
N	66 Countries				65 Countries			
Observations	47197				46961			
Marginal R^2 / Conditional R^2	0.079 / 0.181				0.230 / 0.322			
AIC	78059.805				70746.166			

Table S7. Weighted linear multilevel regression predicting policy support for European winter storms (random intercepts across countries)

	**Step 1: Exposed population**				**Step 2: Moderation analyses**			
Predictors	*Beta*	*SE*	*Statistic*	*p*	*Beta*	*SE*	*Statistic*	*p*
Intercept	2.444	0.015	164.292	**<.001**	2.44	0.015	167.021	**<.001**
Gender (male)	0	0.002	-0.144	.885	0.01	0.002	5.630	**<.001**
Age	-0.015	0.002	-8.636	**<.001**	-0.022	0.002	-13.084	**<.001**
Education (tertiary)	0.038	0.002	19.259	**<.001**	0.033	0.002	17.771	**<.001**
Income	0.01	0.002	5.125	**<.001**	0.008	0.002	4.210	**<.001**
Residence place (urban)	0.027	0.002	14.239	**<.001**	0.022	0.002	12.574	**<.001**
Political orientation (right)	-0.056	0.002	-24.871	**<.001**	-0.039	0.002	-18.199	**<.001**
Political orientation (conservative)	-0.042	0.002	-18.514	**<.001**	-0.031	0.002	-14.352	**<.001**
Religiosity	0.024	0.002	12.009	**<.001**	0.016	0.002	8.878	**<.001**
Exposed population to winterstorms	-0.013	0.016	-0.827	.412	-0.011	0.015	-0.733	.466
Subjective attribution					0.148	0.002	80.663	**<.001**
Exposed population to winterstorms * Subjective attribution					0.009	0.002	4.872	**<.001**
Exposed population to winterstorms * Income					-0.003	0.002	-1.833	.067
Exposed population to winterstorms * Residence (urban)					0.002	0.002	1.055	.291
**Random Effects**								
σ^2	0.1				0.09			
τ_{00}	0.01				0.01			
ICC	0.11				0.12			
N	66 Countries				65 Countries			
Observations	47197				46961			
Marginal R^2 / Conditional R^2	0.079 / 0.182				0.228 / 0.321			
AIC	78059.482				70776.196			

Table S8. Weighted linear multilevel regression predicting mean level of subjective attribution across events (random intercepts across countries)

	Mean subjective attribution			
Predictors	*Beta*	*SE*	*t*	*p*
Intercept	3.887	0.039	99.468	**<.001**
Gender (male)	-0.074	0.005	-16.445	**<.001**
Age	0.029	0.004	6.879	**<.001**
Education (tertiary)	0.044	0.005	9.353	**<.001**
Income	0.014	0.005	3.120	**.002**
Residence place (urban)	0.031	0.004	6.934	**<.001**
Political orientation (right)	-0.132	0.005	-25.085	**<.001**
Political orientation (conservative)	-0.092	0.005	-17.352	**<.001**
Religiosity	0.053	0.005	11.352	**<.001**
**Random Effects**				
σ^2	0.57			
τ_{00} COUNTRY_NAME	0.1			
ICC	0.14			
N COUNTRY_NAME	67			
Observations	48292			
Marginal R^2 / Conditional R^2	0.065 / 0.199			
AIC	163225.172			

Table S9. Mean and standard deviation for extreme weather event variables.

**Extreme weather events**	**Mean**	**SD**
Droughts	0.067	0.102
Heatwaves	0.996	0.028
Heavy precipitation	0.999	0.003
Floods	0.077	0.079
Tropical Cyclones	0.089	0.223
Wildfires	0.291	0.252
European winterstorms	0.104	0.194

Note: A mean value of 1 corresponds to 100% exposed population, while a value of 0 corresponds to 0% exposed population.

Table S10. Reliability (omega) of subjective attribution scale across 67 countries in ascending order

Country	Omega		Country	Omega
Kenya	0.744		Japan	0.895
Uganda	0.752		New Zealand	0.896
Cameroon	0.761		Colombia	0.900
Bangladesh	0.773		Georgia	0.902
Ghana	0.778		Bulgaria	0.904
Côte d'Ivoire	0.786		Serbia	0.905
Morocco	0.790		Mexico	0.906
Nigeria	0.793		Russia	0.906
Ethiopia	0.800		Romania	0.912
Bolivia	0.803		Israel	0.913
India	0.814		Italy	0.914
Botswana	0.817		Uruguay	0.916
Congo DR	0.821		Switzerland	0.919
Indonesia	0.823		Denmark	0.921
Chile	0.823		France	0.921
Peru	0.823		Greece	0.921
China	0.832		United Kingdom	0.923
Egypt	0.835		Finland	0.926
Nicaragua	0.842		Poland	0.926
Kazakhstan	0.843		Portugal	0.927
Hungary	0.844		Slovakia	0.928
Ukraine	0.846		Belgium	0.929
Philippines	0.852		Cyprus	0.931
Malaysia	0.854		Australia	0.933
Hong Kong	0.856		Netherlands	0.936
South Korea	0.861		Austria	0.936
Türkiye	0.862		Canada	0.938
Taiwan	0.866		Slovenia	0.938
Brazil	0.876		Norway	0.939
South Africa	0.881		Germany	0.942
Ireland	0.885		Spain	0.944
Costa Rica	0.885		Sweden	0.945
Czech Republic	0.887		United States	0.949
Argentina	0.888			

Table S11. Reliability (omega) of aggregate policy support scale across 66 countries in ascending order

Country	Omega	Country	Omega
Ukraine	0.404	Slovenia	0.597
Côte d'Ivoire	0.440	Sweden	0.602
Australia	0.477	Slovakia	0.617
New Zealand	0.484	Nicaragua	0.618
Kenya	0.489	Botswana	0.619
Russia	0.491	Germany	0.623
Switzerland	0.509	Canada	0.624
Belgium	0.514	Morocco	0.624
Romania	0.528	Colombia	0.628
Austria	0.531	Israel	0.631
Cameroon	0.533	Serbia	0.632
Egypt	0.536	Ireland	0.639
Hong Kong	0.542	Nigeria	0.642
Costa Rica	0.545	Italy	0.643
India	0.552	Indonesia	0.643
Bulgaria	0.554	Uganda	0.648
Taiwan	0.555	Mexico	0.651
China	0.559	Spain	0.656
Czech Republic	0.560	Kazakhstan	0.659
Finland	0.565	Ghana	0.660
Netherlands	0.570	United Kingdom	0.661
Chile	0.570	Brazil	0.667
France	0.572	Uruguay	0.674
Norway	0.578	Albania	0.696
South Africa	0.584	Georgia	0.701
South Korea	0.588	Ethiopia	0.702
Poland	0.590	Congo DR	0.705
Philippines	0.591	United States	0.710
Bangladesh	0.592	Bolivia	0.714
Denmark	0.592	Hungary	0.717
Japan	0.595	Türkiye	0.732
Greece	0.595	Portugal	0.736
Cyprus	0.595	Peru	0.753

Table S12. Polychoric exploratory factor analysis (EFA) with items measuring climate policy support

	**Factor 1 "Green transition"**	**Factor 2 "Taxes"**
Raising carbon taxes on fossil fuels (e.g., gas or goal)		0.90
Expanding infrastructure for public transportation	0.56	
Increasing the use of sustainable energy such as wind and solar energy	0.78	
Protecting forested and land areas	0.85	
Increasing taxes on carbon intense foods		0.78

Note: EFA was performed with unweighted least squares factoring and promax oblique rotation. Factor loadings < .20 are omitted.

Table S13. Definitions of extreme weather events and data sources.

**Hazard**	**Years**	**Hazard variable/definition**	**Data source**	**Additional information**
**Drought**	1980-2005	led = annual land area fraction exposed to drought as hazard variable in CLIMADA; Entire grid cell if monthly soil moisture falls below the 2.5th percentile (Klein Goldewijk et al., 2017) of the preindustrial baseline distribution for at least seven consecutive months.	ISIMIP 2a/Lange et al. (2020)	Output of 6 (historical) global hydrological models x 4 climate models (GCMs) = 24 ensembles were used to calculate the multimodel median; "histsoc" (varying direct human influences in the historical period).
**River Flood**	1980-1999	Maximal annual river flood depth at each location as hazard variable in CLIMADA; Grid cell fraction with depth exceeding 1m (used for instance as threshold for displacement Kam et al. (2021)).	CLIMADA API / ISIMIP 2a / Sauer et al. (2021)	46 ensembles for historical were used (combination of GCMs and hydrological models) to calculate the multimodel median.
**Heatwaves**	2000-2019	Increase in number of heat days per year. A heat day is defined as daily mean temperature exceeds the 99th percentile of the reference period 1980-1999 and is warmer than 15°C. To only display the increase, the expected 73 (20*365*0.01) days exceeding the 99th precentile are subtracted.	ERA5 reanalysis data (Hersbach et al., 2020)	

**Heavy precipitation**	2000-2019	Increase in number of heavy precipitation days per year. A heavy precip day is defined as daily mean precipitation that exceeds the 99th percentile of the reference period 1980-1999 and is more intense than 5mm. To only display the increase, the expected 73 (20*365*0.01) days exceeding the 99th precentile are subtracted.	ERA5 reanalysis data (Hersbach et al., 2020)	
**Wildfires**	2000-2020	2000-2020<br>Satellite imagery derived thermal anomaly; annual maximum; grid cell counts as affected if temperature > 300K Lüthi et al. (2021)	CLIMADA API / Lüthi et al. (2021) (FIRMS/MODIS data)	Output of 4 GCMS x 5 vegetation models = 20 ensembles were used to calculate the multimodel median.
**Tropical cyclones**	2000-2019	People affected by Cat. 1 tropical cyclones and stronger (> 33m/s wind speed).	IBTrACS/Knapp et al. (2010)	Observed tropical cyclones
**European Winterstorms**	1994-2014	People affected by windspeeds of tropical Cat 1 category or stronger (to insure compatibility with TCs). Only modelled for countries:<br>'ALB', 'AUT', 'BEL', 'DNK', 'FIN', 'FRA', 'DEU', 'GRC', 'HUN', 'IRL', 'ITA', 'MAR', 'NLD', 'POL', 'ROU', 'SVK', 'SVN', 'ESP', 'SWE', 'CHE', 'TUR', 'UKR', 'GBR'	CLIMADA API / Röösli (2021) (WISC data)	Observed winter storms. Data aggregated over 3 days, so for instance the storms Lothar on 26.12.1999 and Martin on 27.12.1999 are counted only as one.

The following members of the TISP Consortium are not listed as co-authors:

Marlene Altenmüller, Richard Amoako, Cornelia Betsch, Apurav Yash Bhatiya, Steven De Peuter, Tom W. Etienne, Simon Fuglsang, Winfred Gatua, Mario Gollwitzer, Gina M. Grimshaw, Lelia N. Hawkins, Maho Ishibashi, Younes Jeddi, Zhangir Kabdulkair, Jo-Ju Kao, Eleni A. Kyza, Hugo Mercier, Julia Metag, Iryna Mudra, Jaime Palmer-Hague, Myron A. Penner, Jan Pfänder, Cintia Refojo Seronero, Simone Rödder, Philipp Schmid, Bermond Scoggins, Amena Sharaf, Justin Sheria Nfundiko, Olivier Standaert, Gert Storms, Christiana Varda, Steven Verheyen, Iain Walker, Marcel Weber, Florian Wintterlin, Rolf A. Zwaan

Reference

Auguie, A., Goldie, J., & Thériault, R. (2024). *ggflags: Plot flags of the world in ggplot2* (Version R package version 0.0.4). https://github.com/rensa/ggflags
